# Rate of Formation of Industrial Lubricant Additive Precursors from Maleic Anhydride and Polyisobutylene

**DOI:** 10.1021/acs.oprd.2c00207

**Published:** 2022-08-30

**Authors:** Jessica Streets, Nicolas Proust, Dixit Parmar, Gary Walker, Peter Licence, Simon Woodward

**Affiliations:** † GSK Carbon Neutral Laboratories for Sustainable Chemistry, University of Nottingham, Triumph Road, Nottingham NG7 2TU, U.K.; ‡ The Lubrizol Corporation, Wickliffe, Ohio 44092, United States; § The Lubrizol Corporation, Hazelwood, Derby DE56 4AN, U.K.

**Keywords:** kinetics, ene reaction, thermal, mechanism, energetics, lubricant

## Abstract

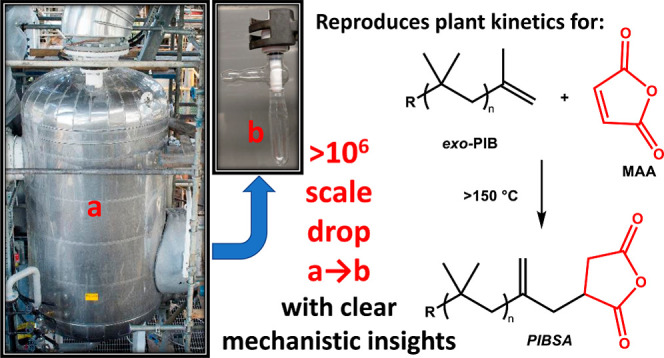

The Alder-ene reaction of neat polyisobutylene (**PIB**) and maleic anhydride (**MAA**) to produce the industrially important lubricant additive precursor polyisobutylene succinic anhydride (**PIBSA**) is studied at 150–180 °C. Under anaerobic conditions with [**PIB**] ∼ 1.24 M (550 g mol^–1^ grade, >80% *exo* alkene) and [**MAA**] ∼ 1.75 M, conversion of *exo*-**PIB** and **MAA** follows second-order near-equal rate laws with *k*_obs_ up to 5 × 10^–5^ M^–1^ s^–1^ for both components. The *exo*-alkene-derived primary product **PIBSA-I** is formed at an equivalent rate. The less reactive olefinic protons of *exo*-**PIB** also react with **MAA** to form isomeric **PIBSA-II** (*k*_obs_ up to 6 × 10^–5^ M^–1^ s^–1^). Some *exo*-**PIB** is converted to *endo*-**PIB** (containing trisubstituted alkene) in a first-order process (*k*_obs_ ∼ 1 × 10^–5^ s^–1^), while **PIBSA-I** is difunctionalized by **MAA** to bis-**PIBSA**s very slowly. The **MAA-** and **PIB-**derived activation parameter Δ*G*^‡^(150 °C) 34.3 ± 0.3 kcal mol^–1^ supports a concerted process, with that of **PIBSA-I** suggesting a late (product-like) transition state.

## Introduction

**Scheme 1:**
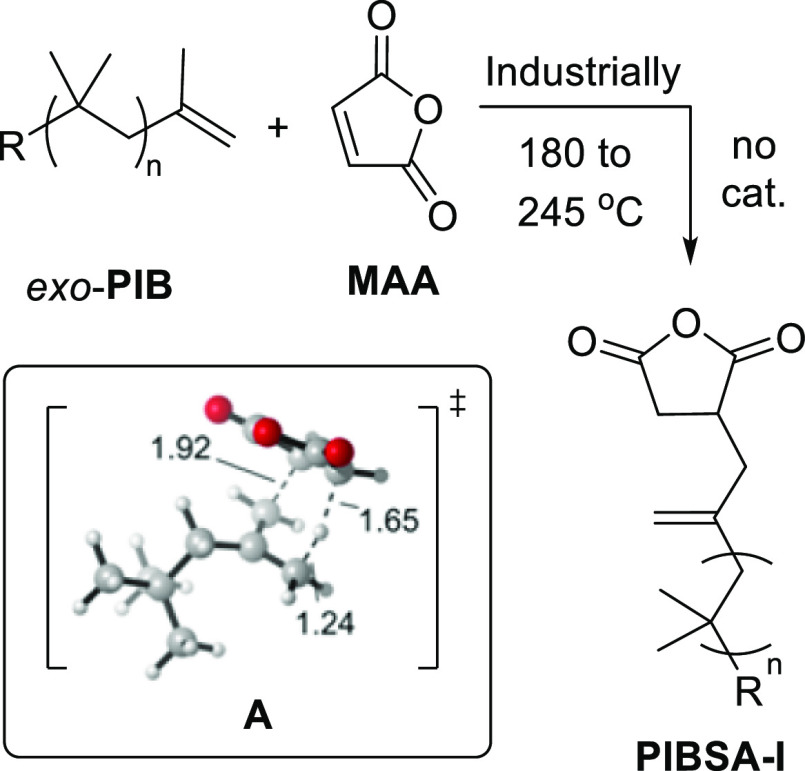
Industrial Preparation of **PIBSA-I** (R = Polymer Chain) and Calculated^7^ Model ene Transition State (**A**, R = *t*-Bu), Showing Interatomic Distances in Å

Lubricating oils and emulsifiers are important global additives with a myriad of technological applications. The total global dispersant market (2021) has been valued at >$6 billion.^1^ Polyisobutylene succinic anhydride (**PIBSA**, Scheme 1) occupies a critical market position in the automotive sector and is manufactured on bulk scales (>10^4^ tons per year) with an estimated 2022 value of ca. $1.5 billion.^2^ Presently, most **PIBSA** is attained by a direct thermal reaction of α-olefin-terminated polyisobutylene (**PIB**) and maleic anhydride (**MAA**) (Scheme 1).^3−5^ This reaction is believed to proceed via a classical (uncatalyzed) Alder-ene reaction^3,6^ and requires high temperatures (>150 °C) and long reaction times (>20 h) even when the neat reagents are combined.

**Scheme 2:**
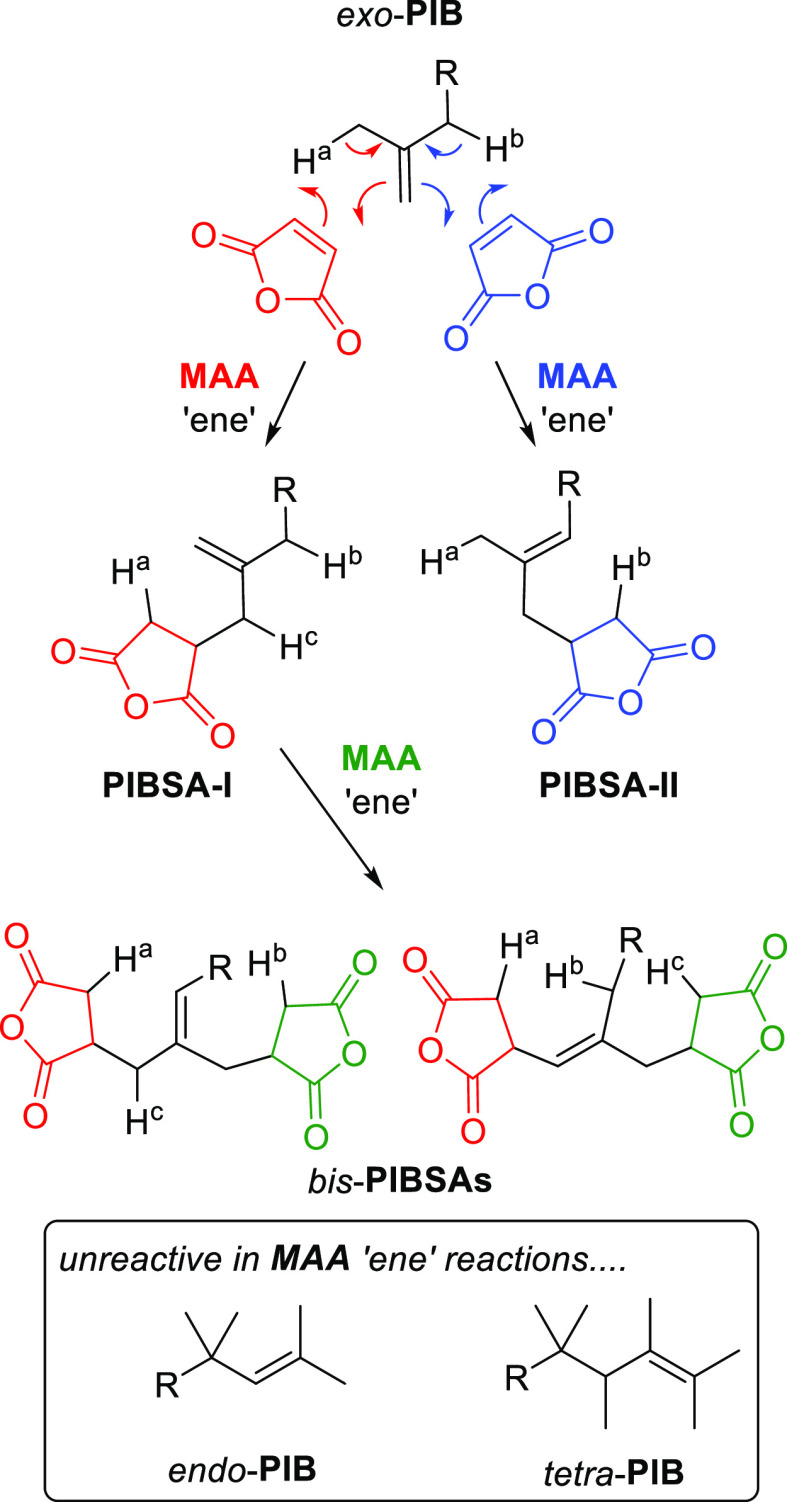
Full Product Distribution of Species Formed in Industrial **PIBSA** Production (R = Polymer Chain)

Although the reaction is industrially valuable, the vigorous reaction conditions associated with the industrial process have largely precluded quantitative mechanistic rate investigations. Such investigations could offer insights into how to reduce the present demanding reaction times and temperatures used in current generation industrial **PIBSA** plants. As it is produced at bulk scales under vigorous conditions, small increases in the reaction efficiency disproportionally improve the environmental credentials of the reaction in terms of reduced carbon footprint and related UN sustainable development goals.^8^ Even in the most general sense, studies of the kinetics of the Alder-ene reaction are surprisingly limited, and all of these have been carried out under dilute solvent-based conditions, which are unrepresentative of the true industrial process.^6,9,10^ Additionally, there are ad hoc observations from production runs that the **PIB**/**MAA** Alder-ene reaction is rather sensitive to the presence of traces of oxygen (or other radical promotors), leading to the formation of alternative products via different mechanisms.^3,4,6,11^ A recent (2021) computational study modeled a concerted Alder-ene [4 + 2] pericyclic transition state (**A**) between **MAA** and 2,4,4-trimethylpent-1-ene (*t*-BuCH_2_C(=CH_2_)Me), as a surrogate for the end of a **PIB** chain (Scheme 1).^7^ This study provided a calculated Gibbs activation energy of 36.6 kcal mol^–1^ (at 150 °C) with an associated enthalpy change (Δ*H*^‡^) of 15.8 kcal mol^–1^. A similar activation barrier (36.7 kcal mol^–1^) has been calculated (2021) for the uncatalyzed ene reaction between propene and but-3-en-2-one.^12^ Both of these papers^7,12^ suggest that a significant rate acceleration should be realized in the presence of AlCl_3_ due to Lewis acid catalysis. We were, therefore, interested in contrasting the theoretical energy barrier for the uncatalyzed reaction to those attained experimentally under conditions that closely simulate the industrial process. Herein, we describe a detailed kinetic study of the direct thermal reaction of **PIB** with **MAA** and comment briefly on the effect of small amounts of AlCl_3_ on the reaction. The true industrial thermal “ene” synthesis is more complex than the headline summary of Scheme 1. A cascade of competing processes (Scheme 2) occurs during the overall production of **PIBSA**.

Typically, high vinylidene **PIB** is used in industrial synthesis, containing >80% α-olefin-terminated **PIB** (*exo*-**PIB**), with the remaining composition being β-olefins (*endo*-**PIB**, >10%) and some tetra-substituted alkenes (tetra-**PIB**). These latter two alkenes are not active in Alder-ene chemistry. However, the allylic protons of *exo*-**PIB** (H^a^ and H^b^ in Scheme 2) react with **MAA** to generate isomeric **PIBSA-I** and **PIBSA-II**, respectively. Further reaction of equivalent allylic protons (labelled H^b^ and H^c^) within **PIBSA-I** lead to the formation of the bis-**PIBSA** structures shown. Fortunately, although conformation-induced peak broadening and some overlaps occur, diagnostic ^1^H NMR peaks of all the species within Scheme 2 are available and assigned from the literature precedent,^3^ allowing their complete quantification as a function of time. Tetra-**PIB** cannot be monitored by NMR during **PIBSA** synthesis due to the overlap of assigned NMR peaks with those of product **PIBSAs**.

## Results and Discussion

**Figure 1:**
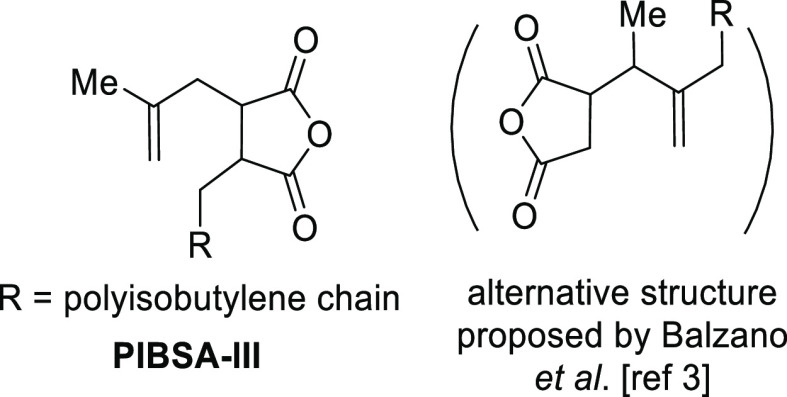
Structure of **PIBSA-III**, a common impurity in aerobic compromised **PIBSA** generation (see also the Supporting Information).

Monitoring of reactions of neat **PIB** and **MAA** is complicated by three factors: (i) **MAA** is only readily soluble in **PIB** above ca. 100 °C and separates stochastically on rapid cooling (invalidating aliquot sampling); (ii) **MAA** is volatile and lost to the reaction headspace under the reaction conditions, causing mass balance/reaction homogeneity issues; (iii) competing radical-based reaction pathways are easily^3^ promoted by trace amounts of oxygen (air), leading to alternative byproducts. Preliminary studies showed that aliquot sampling from a single vessel led to very poor reproducibility/induction periods. In standard glassware, the major (but batch-dependent) product was **PIBSA-III**, assigned by us as the structure given in Figure 1, on the basis of our NMR data. A regioisomeric structure has also been proposed by Balzano and co-workers,^3^ but in either case, its formation is favored by radical initiators, especially trace oxygen.^11^ Such issues have previously prevented accurate kinetic analyses of this reaction, even in the presence of radical inhibitors.^6^

Issues (i)–(iii) were overcome using minimal headspace glass ampoules with Young’s tap seals and thorough degassing (see the Experimental Section). By accounting for the different *t*_1_ relaxation values of 550 g mol^–1^**PIB** and its derivatives versus lighter **MAA** and nitrobenzene NMR standard used, it was possible to obtain quantitative composition-time information from ^1^H NMR spectra (see the Supporting Information for details) for all **PIB**-containing components. A small series of experiments were conducted to model the background variation in the distribution of **PIB** structures, in the absence of **MAA**, at 150, 165, and 180 °C for 4, 8, 15, 20, and 24 h. No statistically significant variation in the composition occurred, indicating that **PIB** is stable to the reaction conditions in the absence of other components.

Kinetic investigations of the reaction of **PIB** with **MAA** were then completed at 150, 160, 165, 170, and 180 °C, and the composition–time data was extracted by NMR. The nominal molarity of each species was calculated from each spectrum, accounting for the density of **PIB** observed at the experimental temperatures. These results are consistent and reproducible for **PIB** and **PIBSA** species for identical runs (±1–2%), but the quantity of **MAA** present was variable. This variation is due to the poor solubility of **MAA** in **PIB** at room temperature. Although there was no loss of reaction mass, the consumption of **MAA** cannot be monitored by our NMR approach due to its irreproducible precipitation in **PIB** mixtures. The **MAA** content could be quantified by gas chromatography (GC) after solubilizing the total ampoule contents in CH_2_Cl_2_, although this procedure had a lower reproducibility (ca. 5% error).

The experimental concentration–time data are found to best fit the integrated rate laws (1) to (4)^13^ for **MAA**, **PIB**, and **PIBSA** species when using nonlinear least squares regression to determine rate constant values (*k*_obs_) and goodness-of-fit.^14−16^ Statistical analysis of each data set was carried out using SolverStat.^17^ This indicated that the second-order near equal concentrations regime best fitted the decay of *exo*-**PIB**, eq 1, and **MAA**, eq 2, and growth of **PIBSA-I** and **PIBSA-II**, eq 3, where Δ_0_ = [MAA]_0_ – [PIB]_0_.^13^ Growth of *endo*-**PIB** is best modeled using first-order kinetics, eq 4. The observed rate constants, *k*_obs_, derived from fitted experimental data for the decay or formation of each species monitored are given in Table 1. Pseudo-first-order rate constants, *k*_1_, necessary for subsequent reaction parameter calculations were obtained by (i) multiplication of *k*_obs_ (M^–1^ s^–1^) by [**MAA**]_0_ for *exo*-**PIB**, **PIBSA-I**, and **PIBSA-II**, (ii) multiplication of *k*_obs_ (M^–1^ s^–1^) by [**PIB**]_0_ for **MAA**, and (iii) division of *k*_obs_ (M s^–1^) by [**MAA**]_0_ for bis-**PIBSAs**. *Endo*-**PIB** forms under first-order conditions, so *k*_obs_ = *k*_1_ (s^–1^). The worst errors were associated with the formation of bis-**PIBSA**s and *endo*-**PIB** due to their low concentrations, especially at lower temperatures. Interestingly, the generation of *endo*-**PIB** is marginally faster in the presence of **MAA** and **PIBSAs** than in the presence of **PIB** alone. We attribute this to the adventitious generation of trace acid catalyst for C=C bond isomerization.
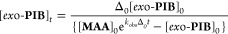
1
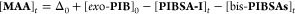
2
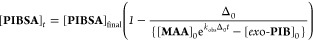
3
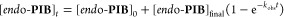
4

**Table 1:** Rate Constants for the Processes of Scheme 2^a^

process	temp (°C)	*k*_obs_ (M^–1^ s^–1^)	*k*_1_ (s^–1^)
consumption of **MAA**	150	8(3) × 10^–6^	1.0(4) × 10^–5^
	160	3(1) × 10^–5^	4(1) × 10^–5^
	165	2(1) × 10^–5^	3(1) × 10^–5^
	170	4(1) × 10^–5^	5(1) × 10^–5^
	180	5.0(1) × 10^–5^	6(1) × 10^–5^
consumption of *exo***-PIB**	150	3.9(6) × 10^–6^	7(1) × 10^–6^
	160	1.6(3) × 10^–5^	2.8(4) × 10^–5^
	165	1.7(2) × 10^–5^	3.0(4) × 10^–5^
	170	2.6(3) × 10^–5^	4.5(6) × 10^–5^
	180	4.1(6) × 10^–5^	7(1) × 10^–5^
formation of **PIBSA-I**	150	3(2) × 10^–6^	5(3) × 10^–6^
	160	5(4) × 10^–6^	9(6) × 10^–6^
	165	1.4(4) × 10^–5^	2.5(6) × 10^–5^
	170	2.4(8) × 10^–5^	4(1) × 10^–5^
	180	6(1) × 10^–5^	1.1(3) ×10^–4^
formation of **PIBSA-II**	150	1.6(6) × 10^–5^	3(1) × 10^–5^
	160	5(4) × 10^–6^	8(7) × 10^–6^
	165	1(2) × 10^–6^	1(4) × 10^–6^
	170	2.1(5) × 10^–5^	3.6(8) × 10^–5^
	180	6(1) × 10^–5^	1.0(2) × 10^–4^

a For the formation of *endo*-**PIB** and bis-**PIBSAs**, see the Supporting Information. The number in parentheses is the standard deviation in the preceding digit.

Exact forms of the integrated rate law equation for two consecutive second-order reactions (to simulate the formation of bis-**PIBSA**) are not available.^18^ However, due to the significant excess of *exo*-**PIB** and **MAA** compared to bis-**PIBSA**, this reaction became near zeroth order and was modeled as such. Alternative attempts to extract the composite second-order rate constants through Excel-based simulation methods^19^ were unsuccessful.

Owing to the occurrence of *exo* to *endo* alkene isomerization, the formation of both **PIBSA-I** and **-II** structures from *exo*-**PIB** and the consumption of **PIBSA-I** to form bis-**PIBSAs** at higher temperatures, all measured components were treated separately. The rate of consumption of **MAA** or **PIB** somewhat exceeds the rate of formation of **PIBSA-I**. This difference is attributed to the accelerated formation of minor species not detected by the NMR assay as the temperature rises and agrees with mass balance loss discussed later. This is also in accord with the minor mass balance issues sometimes noted in plant scale operations over time. A good correlation of the models of eqs 1–4 is attained, with most individual data fits in the range 0.79–0.96 (*R*^2^). This is acceptable and still generates meaningful data, especially considering the challenging sampling procedure required. The most significant sources of error are in the GC-based measurement of [**MAA**]_*t*_ and in the determinations of [*endo*-**PIB**]_t_ and [bis-**PIBSA**]_*t*_ (particularly the latter two, which are only present at low concentrations). Attempts to extend our study above 180 °C were not successful with our present setup.

**Figure 2:**
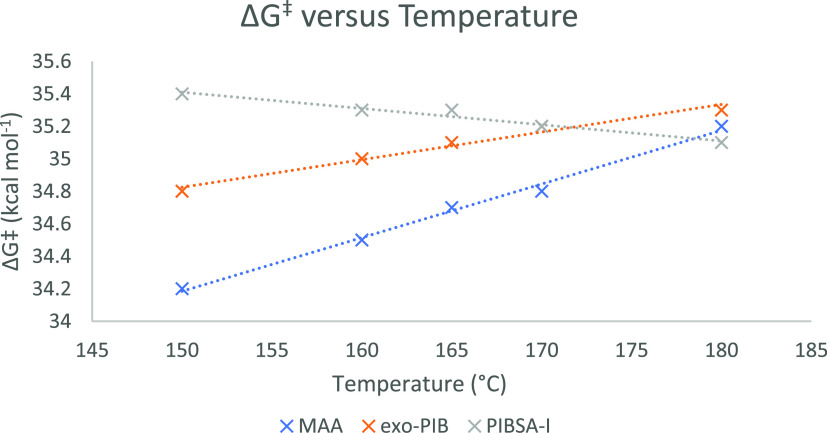
Overall Δ*G*^‡^ vs temperature for transformations of Scheme 2 based on **MAA** and *exo*-**PIB** consumption and **PIBSA-I** formation.

The Eyring–Polanyi equation (eq 5) allows estimation of the Gibbs free energy of activation (Δ*G*^‡^) for a reaction and its deconvolution into Δ*H*^‡^ and Δ*S*^‡^ (see the Supporting Information). Plotting the derived Δ*G*^‡^ values for **MAA**, *exo*-**PIB**, and **PIBSA-I** versus temperature is informative (Figure 2). Both **MAA** and *exo*-**PIB** show increasing Δ*G*^‡^ with increasing temperature. Such behavior either indicates significant ordering in the transition state (i.e., a strong negative Δ*S*^‡^ term) or that additional reaction manifolds (requiring higher Δ*G*^‡^) are becoming available as the reaction temperature increases. Conversely, the Δ*G*^‡^ values attained from the rate of **PIBSA-I** formation fall as temperature rises. Even allowing for the experimental error, the difference between Δ*G*^‡^ of the starting materials and product is beyond the error bar.
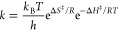
5

While the experimental Δ*G*^‡^(150 °C) values from **MAA**, *exo*-**PIB**, and **PIBSA-I** (34.1 ± 1.5, 34.8 ± 2.2, and 35.4 ± 2.2 kcal mol^–1^, respectively), compare well to those (36.6 kcal mol^–1^) derived from density functional theory (DFT) studies,^7,12^ the relative slopes of Figure 2 point to a more complicated picture. Table 2 presents the Eyring-Polanyi Δ*H*^‡^ and Δ*S*^‡^ values deconvoluted from the **MAA**, *exo*-**PIB**, and **PIBSA-I** Δ*G*^‡^ data. As no literature Δ*H*^‡^ or Δ*S*^‡^ experimental values are available for individual components of the Alder-ene reaction of **PIB** and **MAA**, Arrhenius plots of each dataset (**MAA**, *exo*-**PIB**, and **PIBSA-I**) were also made (see the Supporting Information) to determine the activation energy (*E*_a_) from each of these components (eq 6 and Table 2).
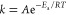
6

**Table 2:** Δ*H*^‡^, Δ*S*^‡^, and *E*_a_ Values from the **MAA**, *exo*-**PIB** and **PIBSA-I** Rate Data^a^

reaction process	Δ*H*^‡^ (kcal mol^–1^)	Δ*S*^‡^ (eu)	*E*_a_ (kcal mol^–1^)
consumption of **MAA**	21(7)	–33(15)	21.6(66)
consumption of *exo***-PIB**	28(5)	–16(11)	29.0(47)
formation of **PIBSA-I**	40(4)	11(9)	40.9(40)

a The number in parentheses is the standard deviation in the preceding digit.

Literature activation energies (*E*_a_) of all published Alder-ene reactions using maleic anhydride are presented in Table 3, together with how the values were attained.^9−11^ No literature value exists for the **PIB** and **MAA** system for direct comparison; Martuano has attempted this previously but was unable to reproducibly quantify the reaction components using high-performance liquid chromatography or GC methods.^6^ The activation energy of the ene reaction between **MAA** and polypropylene (*M*_n_ ∼2010 g mol^–1^, *M*_w_ ∼10,300 g mol^–1^) has been calculated as 22.0 kcal mol^–1^ from an IR-derived rate of consumed **MAA**.^10^ These studies^6,9,10^ all conclude that **MAA**-ene reactions are first order with respect to both alkene and enophile and second order overall, in line with our own findings. However, as far as we can determine, no previous Eyring analysis of all of the components of an Alder-ene synthesis has previously been undertaken, even though the reaction is 80 years old. While the **PIB** system can be expected to have a slower rate due to the increased steric bulk of the polymeric alkene, the (reproducible) activation parameters of **PIBSA-I** (Table 2) are not in accord with the large-Δ*S*^‡^ term seen for classic pericyclic reactions. One potential rationale for the data in Table 2 is that the **MAA** and **PIB** consumption data is “contaminated” by competing higher energy processes. In line with this, some deviation between the calculated reaction composition data and the observed amounts of **PIB** and **MAA** is observed. At 150 °C, 11% of the mass balance is unaccounted for after 24 h. At 180 °C, this figure rises to ca. 30%. We can detect no mass loss from our reactions, implying that depolymerization of **PIB** to isobutylene is not an issue. This indicates the production of additional product(s) undetected by the NMR and GC assays. These products must be insoluble in CDCl_3_ or be sufficiently line broadened to not have clear NMR peaks. Gel permeation chromatography (GPC) analysis additionally did not reveal any more information, and the mass balance loss does not correlate to the IR signal that has been assigned to poly(maleic anhydride) species.^10^ The undetected byproducts are most likely high-molecular-weight solid polymers.

**Table 3:** Available Kinetic Data for Alder-ene Reactions Using **MAA**

alkene	*E*_a_ (kcal mol^–1^)	Δ*S*^‡^ (eu)	how determined	conditions	ref
4-phenylbut-1-ene	16.1 ± 0.1	–47.3 ± 0.2	**MAA** data alone; GC method	C_6_H_3_Cl_3_ solution; excess **MAA**; 4% quinol vs [ene]	(6)
2,4-dimethyl-4-phenylpent-1-ene	12.5 ± 0.3	–52.8 ± 0.7	**MAA** data alone; GC method	C_6_H_3_Cl_3_ solution; excess **MAA**; 7% quinol vs [ene]	(6)
C_6_–C_10_ 1-alkenes	21.5 ± 0.7	–36.4 ± 1.1	averaged *k*_2_ from alkene, **MAA** and product data; GC method	C_6_H_4_Cl_2_ solution; 2% quinol vs [ene]	(9)
trans-dec-5-ene	18.1 ± 1.5	–42.6 ± 3.5	averaged *k*_2_ from alkene, **MAA** and product data; GC method	C_6_H_4_Cl_2_ solution; 2% quinol vs [ene]	(9)
allylbenzene	ca. 20	n/a	**MAA** data alone; titration method	C_6_H_4_Cl_2_ solution	(11)
polypropylene	22.0 ± 2.6	n/a	**MAA** data alone; FTIR method	DMF solution; TEMPO (conc. not specified)	(10)

The **PIBSA-I** data in Table 2, if correct, suggests a late (product-like) transition state where the developing C–H bond is already well established. This is in line with the recent (2021) DFT calculations that triggered our investigation.^7,12^ In a final comparison with these *in silico* studies, we tested the efficacy of the Lewis acid catalyst AlCl_3_, which is predicted to provide strong rate acceleration. At loadings of 3–8 mol % (with respect to **PIB**), conversion of **MAA** and **PIB** to **PIBSA-I** and **II** was essentially unaffected compared to the background reaction. The proportion of *endo*-**PIB** increased compared to uncatalyzed conditions as catalyst loading increased. This outcome is in agreement with the literature that suggests evolution of HCl from catalysts accelerates the *exo*-olefin to *endo*-olefin isomerization.^20^ Given the clear calculated drivers for Lewis acid acceleration and the fact that this is a successful strategy in other Alder-ene reactions,^12^ it is likely that the minor byproducts affecting the recorded rate data for **MAA** and **PIB** are also strong sequestering agents for AlCl_3_.

Previous kinetic studies of the ene reaction( see Table 3) have predominately included a radical inhibitor, such as quinol, or a scavenger, such as TEMPO. A smaller series of reactions was conducted with 2% quinol at 165 °C and monitored by our quantitative NMR methods. The rate of consumption of *exo*-**PIB** fell to 1.4(4) × 10^–5^ M^–1^ s^–1^, which is equal to the rate of formation of **PIBSA-I** in the absence of the radical inhibitor in Table 1. No difference in the rate was observed in the presence of 2% quinol and 5% AlCl_3_ after 4 h at 150 °C (data in the Supporting Information).

**Figure 3:**
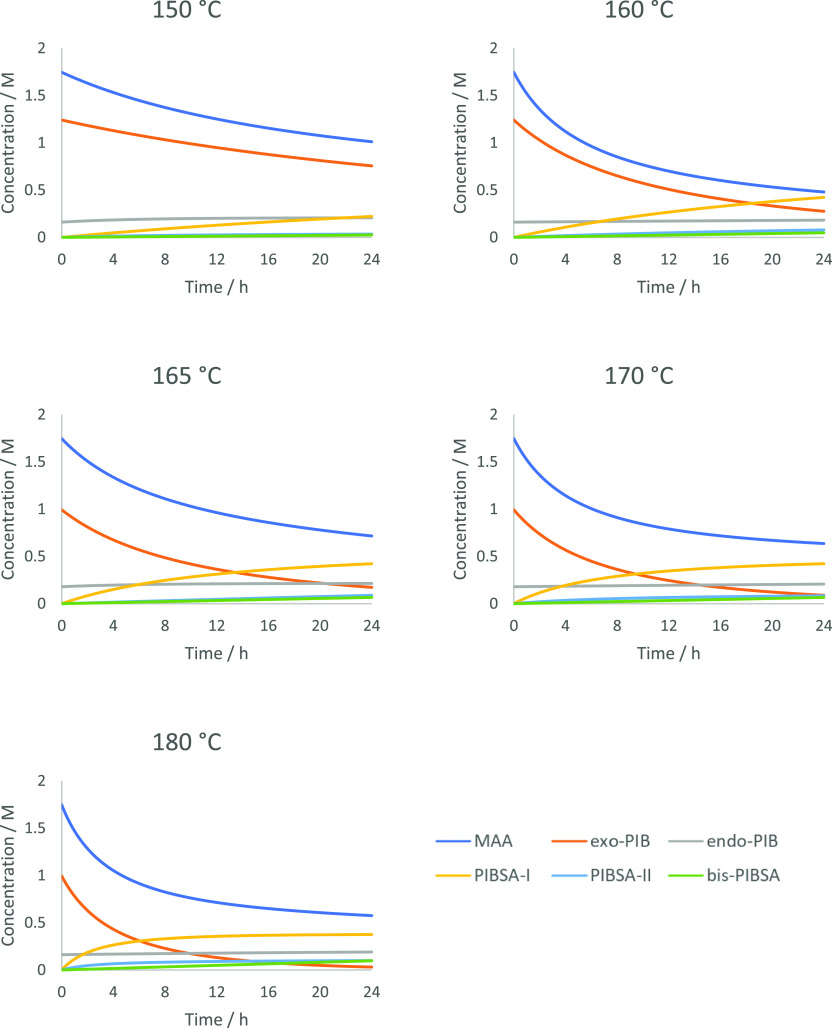
Final simulated reaction composition of the Alder-ene reaction between **PIB** and **MAA** at 150, 160, 165, 170, and 180 °C across 24 h using eqs 1–4 and the data in Table 1.

Despite these underlying factors, the models of eqs 1–4 and the rate constants derived here do provide a good model for the **PIBSA** process. Figure 3 shows the calculated reaction composition across 24 h at each of the temperatures studied. These profiles are in good accord with the reaction profiles seen at industrial scales.

## Conclusions

A kinetic model of the Alder-ene reaction of neat **PIB** and **MAA** to produce the industrially produced lubricant precursor **PIBSA** has been developed. Rate data attained from all observable reaction components between 150 and 180 °C can accurately reproduce bulk plant behavior as a function of temperature.^21^ Detailed extraction of the key kinetic parameters (Δ*G*^‡^, Δ*H*^‡^, Δ*S*^‡^, and *E*_a_) leads to the conclusion that **MAA** and **PIB** are coproducing small amounts of undetected (by NMR, GC, and GPC) byproducts that engender two negative effects. First, this coproduction skews the acquired activation data attained for the process, complicating its analysis, and second, the same byproducts apparently sequester AlCl_3_ that otherwise would be a good catalyst for the process. Kinetic data from the **PIBSA-I** product of the reaction are unaffected by AlCl_3_ and point to a late (product-like) transition state, where C–H bond formation is already appreciably developed, as seen in recent computational models. Understanding these features points to the need to develop catalysts that are active well below current **PIBSA** plant operating temperatures, avoiding inhibition of byproduct formation, but using alternative activation modes for **PIB** and/or **MAA**. Such approaches would allow new optimization strategies for this important reaction and provide a significant opportunity to reduce the manufacturing footprint.

### Experimental Section

High vinylidene 550 g mol^–1^ molecular weight polyisobutylene (**PIB**) used was of an identical grade to that used for industrial lubricant synthesis (Lubrizol). This **PIB** sample contained 80 mol % α-olefins, 15 mol % β-olefins, and 5 mol % tetra-substituted olefins by ^1^H NMR spectroscopy; GPC studies confirmed its molecular weight and indicated a polydispersity of *M*_W_/*M*_n_ = 1.5. **MAA** was commercial (Alfa Aesar), equivalent to that used in the industrial process; its purity was confirmed as >98% by ^1^H NMR spectroscopy.

### Experimental Set-Up

**Figure 4:**
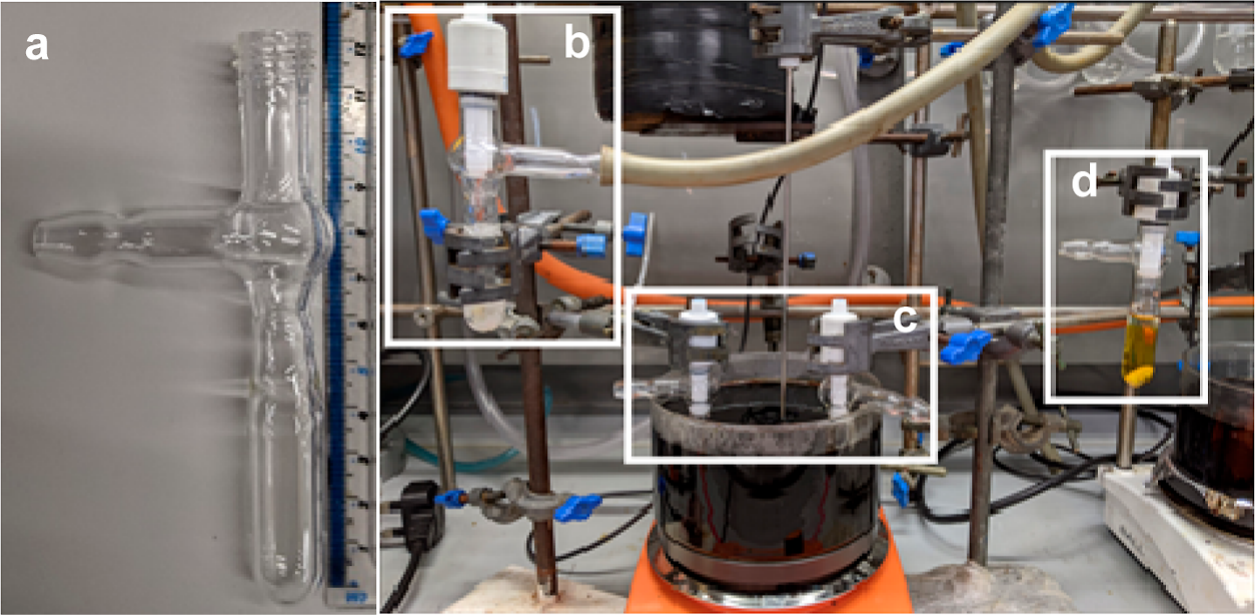
Representative ampoules used in this study: (a) before charging, (b) during degassing, (c) during a typical kinetic run (170 °C), and (d) at the completion of the reaction.

Kinetic runs were conducted using bespoke pressure-resistant glass ampoules with Young’s tap seals (internal diameter, 6 mm; external diameter, 12 mm; height, 120 mm; total volume, 6 mL) (Figure 4). This reaction setup mimics the minimum headspace designs of current industrial **PIBSA** plants and allows multiple duplicate reactions (that give identical conversion-time outputs between batches within ±1–2%) to be set up simultaneously when determining the rates controlling the **PIBSA** cascade (Scheme 2).

### Kinetic Runs

Solid **MAA** (0.816 g, 8.32 mmol, 1.4 equiv), a 10 mm stir bar, and **PIB** (3.270 g, 5.95 mmol, 1.0 equiv) were charged to the ampoule, and Young’s tap was sealed. The reaction mixture was left to settle for 12–16 h to facilitate degassing, which was achieved by 3× vacuum (1 mbar)/N_2_ gas cycles. Young’s tap was closed under a flow of N_2_, and the ampoule was fully submerged in a preheated oil bath (150, 160, 165, 170, or 180 °C), and the kinetics clock started. Sealed reactions were shielded by a blast screen during heated runs. Individual duplicates of the reactions were stopped hourly to provide data over a 24 h window. Owing to the laboratory (covid) open hour restrictions, no data for 11–13 h periods could be collected. To prevent loss of volatile **MAA**, individual reaction samples were cooled to room temperature before the ampoules were opened. Control runs indicated that nominally identically charged ampoule compositions provided identical conversions at given time points (±1–2% conversion). Independent experimental estimates of the densities of **PIB**–**MAA** mixtures in the temperature ranges studied allow the use of molarity, as opposed to molality, units in the kinetic analyses. Amounts of **MAA** were determined by GC and all other species by ^1^H NMR spectroscopy (see the Supporting Information for details). Radical inhibitors were not found to be necessary under these conditions and were not used to avoid potential additional rate data being needed. Conversion in the presence of freshly sublimed AlCl_3_ (3–8 mol % vs PIB) was checked at 150 °C, 4 h and found to be comparable to background conversion within the experimental error (see the Supporting Information). Conversion in the presence of 2% quinol (vs PIB) was calculated at 165 °C at 3, 6, 9, 15, 18, 21, and 24 h and revealed a rate of consumption of *exo*-**PIB** equal to the formation of **PIBSA-I** in the absence of the radical inhibitor (see the Supporting Information).

### Data Analyses

Experimental data were fitted to all kinetic models of eqs 1–4 using Solver Microsoft Excel add-in.^14−16^ Data fits were optimized by nonlinear least squares regression of the sum of ([observed species] – [calculated species])^2^ as a function of *k*_obs_ and where relevant [**PIBSA**]_final_, at fixed [**PIB**]_0_ and [**MAA**]_0_ values. A near equal concentration (second order overall)^13^ rate law gave the best fit to the data based on *R*^2^, except for the isomerization of *exo*-**PIB** to *endo*-**PIB** (which fitted first order) and the formation of bis-**PIBSAs** (which was zeroth order). The SolverStat tool was used to return regression statistics on all coefficients, including the standard deviations and *R*^2^ values (see the Supporting Information).^17^ Derived parameters (*E*_a_, Δ*H*^‡^, Δ*S*^‡^, and Δ*G*^‡^) were calculated from *k*_obs_. The standard deviations for the derived Δ*G*^‡^ values were calculated using eqs 7–11. Full details are given in the Supporting Information.
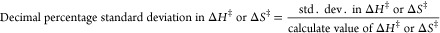
7
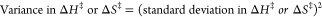
8
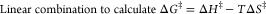
9
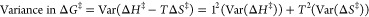
10
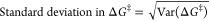
11

All other details and primary data are in the Supporting Information.

## Supporting Information Available

Details of GC and NMR kinetic data acquisition procedures; estimates of safe operating pressures for the glass ampoules used; primary data from all runs; and Eyring–Polanyi and Arrhenius plots (PDF)
