# Supplementary material for: Rate of Formation of Industrial Lubricant Additive Precursors from Maleic Anhydride and Polyisobutylene
Source: Org Process Res Dev. 2022 Aug 30;26(9):2749–55. doi: 10.1021/acs.oprd.2c00207 (PMC9486990; doi:10.1021/acs.oprd.2c00207)
Supplement: Supplementary file 1 — op2c00207_si_001.pdf [file op2c00207_si_001.pdf]

Rate of formation of industrial lubricant additive precursors from maleic anhydride and polyisobutylene

Jessica Streets,[a] Nicolas Proust,[b] Dixit Parmar,[c] Gary Walker,[c] Peter Licence[a] and Simon Woodward[a]*

[a] GSK Carbon Neutral Laboratories for Sustainable Chemistry, University of Nottingham, Triumph Road, Nottingham, NG7 2TU, United Kingdom

[b] The Lubrizol Corporation, Wickliffe, Ohio 44092, United States

[c] The Lubrizol Corporation, Hazelwood, Derby, DE56 4AN, United Kingdom

*Email: simon.woodward@nottingham.ac.uk

■ Supporting Information

1. GC Analyses
2. NMR Analyses
3. Safety Considerations for Glass Ampoules
4. Primary Data from all Runs
5. Eyring-Polanyi Plots and Derived Reaction Parameters
6. Arrhenius Plots and Calculating Reaction Parameters
7. $AlCl_3$ and Quinol Results

1. GC Analyses

The residual **MAA** present in a subset of the ampoule reactions was analysed by GC using a Perkin Elmer Clarus 690 Gas Chromatograph with a HP-5 column 30m x 0.32 mm ID x 1 µm. An injection volume of 2.5 µL, injection temperature of 250 °C and detector temperature of 300 °C were used. All samples were analysed using the temperature programme: 50 °C (2 min), 25 °C min^{-1} up to 250 °C, 50 °C min^{-1} up to 300 °C. For each run the ampoule contents were dissolved in a known amount of CH_2Cl_2, and the **MAA** content of samples attained using a calibration curve constructed from a series of stock standard solutions of known **MAA** concentration. Analysis was completed on a selection of samples across the 24 h period studied at each temperature.

2. NMR Analyses

Quantitative ^{1}H NMR spectra were collected using 0.7 mL of an 0.08 M solution of nitrobenzene in $CDCl_3$, containing a known mass of reaction mixture (ca. 20 mg). Measurements were performed using a 400 MHz Bruker spectrometer at ambient temperature using a relaxation delay of 35 s, 16 scans, a receiver gain of 40.0 and an acquisition time of 8.1920 s. Nitrobenzene, $PhNO_2$, was employed as an external standard; t_1 relaxation measurements confirmed the quantifications were accurate within ±1% per proton. The characteristic integration regions for the NMR standard, starting materials and product are in Table S1. The assignments of the **PIB** and **PIBSA** products follow literature assignments.[1] A representative partial ^{1}H NMR spectrum in the vinylidene region is shown in Figure S1, which also shows the position of the common impurity **PIBSA-III** for reference. Data for **PIBSA-III**: ^{1}H NMR (400 MHz, $CDCl_3$) δ 4.81 (=CH_2), 4.96 (=CH_2) ppm.

Table S1. Characteristic ^1H NMR integration regions for NMR standard, starting material and product species. Protons monitored in each structure are highlighted in bold.

	Structure	Assignments	Protons in Monitored Environment	Integral Region (ppm)	
				Start	End
NMR Standard	$PhNO_2$	$O_2NC_6H_4$-**H (*para*)**	1	7.754	7.655
Reagents	*exo*-**PIB**	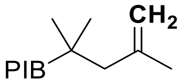	1	4.659	4.617
	endo-**PIB**	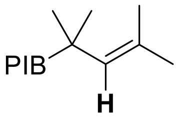	1	5.169	5.101
Products	**PIBSA-I**	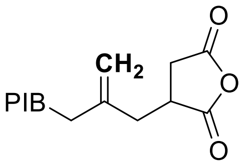	2	4.923	4.890
	**PIBSA-II**	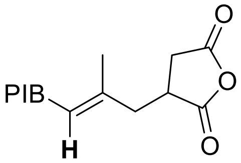	1	5.286	5.270
	bis-**PIBSAs**	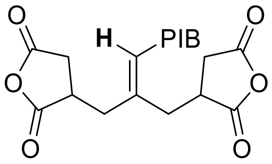 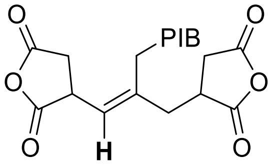	1	5.256	5.212

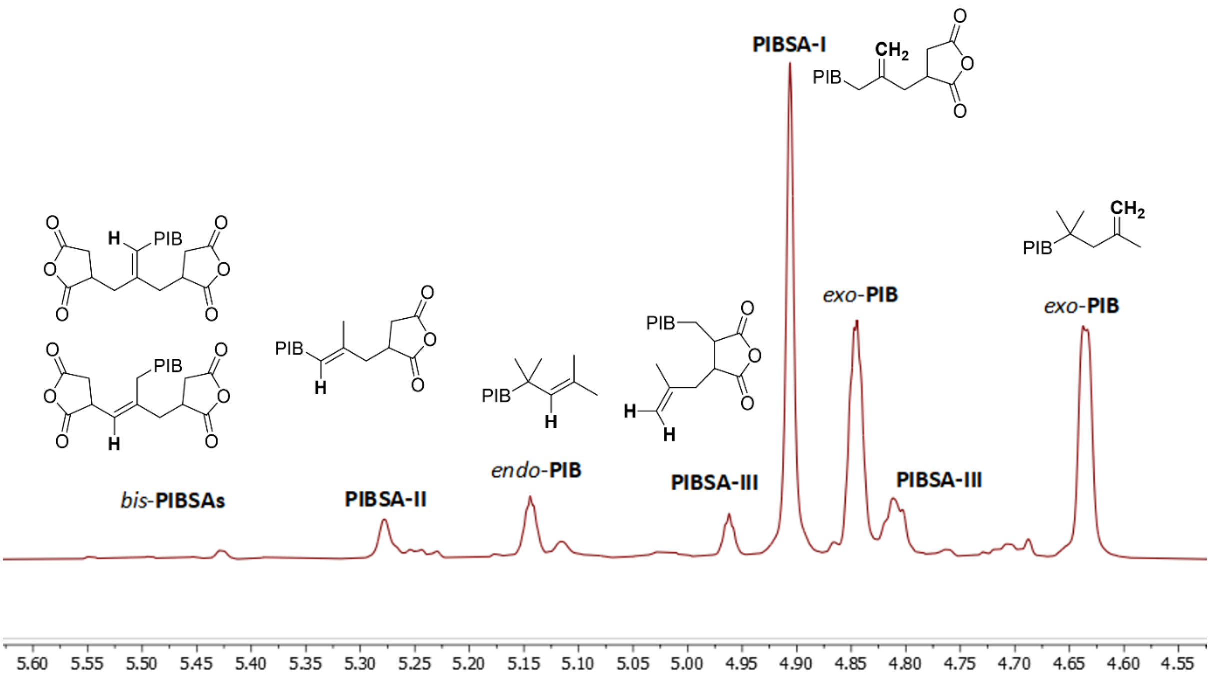

Figure S1. Representative partial ^{1}H NMR spectrum in the vinylidene region and assigned structures. Overlap of minor vinylidene structures from original **PIB** interfere with the *exo*-**PIB** peak at ca. 4.85 ppm and it is thus not diagnostic for quantitative purposes.

Equations used to calculate: mmoles of each structure present in the total reaction mixture (S1), the percentage conversion of *exo*-**PIB** to each **PIBSA** product (S2) and the molarity of each species (S3) are detailed below, where I = integral calculated across ppm range in Table S1. The reaction volume was assumed using the densities of 550 mwt **PIB** (0.77 g mL^{-1} under the conditions studied) and maleic anhydride, **MAA** (1.48 g mL^{-1}).

$$\text{mmoles}_{\text{product}} = I_{\text{product}} \times \frac{\text{mmoles}_{\text{PhNO2}}}{I_{\text{PhNO2}}} \times \frac{\text{reaction mass}}{\text{NMR sample mass}} \qquad \text{(S1)}$$

$$\text{\% Conversion to Product} = \frac{\text{mmoles}_{\text{product}}}{\text{mmoles}_{\text{PIB,t=0}}} \times 100 \qquad \text{(S2)}$$

$$M_{\text{product}} = \frac{\text{mmoles}_{\text{product}}}{\text{Total reaction volume (mL)}} \qquad \text{(S3)}$$

3. Safety Consideration for Glass Ampoules

While all of our studies were made below the boiling point of maleic anhydride (**MAA**), equations (S4) and (S5) were used to estimate the maximum working pressure for the glass ampoules used herein.

$$\text{Pressure (bar)} = \frac{\text{WT} \times 20 \times \text{k/s}}{\text{OD - WT}} \qquad \text{(S4)}$$

In equation (S4): WT = wall thickness in mm (= 2), OD = outer diameter in mm (= 15) and k/s = resistance in N.mm^{-2} (= 7 for the borosilicate glass used in our ampoules).

$$\text{Pressure (Pa)} = (nRT)/V \qquad (S5)$$

In equation (S5): V = volume in m^3 (= 0.0000055), n = moles (= 0.00832), R = gas constant in J mol^{-1} K^{-1} (= 8.314) and T = temperature in K (= 423 to 453). Using equations (S4) and (S5), we estimate our ampoules would be usable to ca. 21.2 atmospheres. No detonations were encountered in our work, but occasional failures of the Young's tap seals did occur after repeated use.

4. Primary Data from all Runs

Table S2. Reaction at 150 °C.

Time / h	**MAA** conc. (M)	*exo*-**PIB** conc. (M)	*endo*-**PIB** conc. (M)	**PIBSA-I** conc. (M)	**PIBSA-II** conc. (M)	*bis*-**PIBSAs** total conc. (M)
0	1.746	0.993	0.161	0.000	0.000	0.000
1		0.946	0.169	0.024	0.012	0.028
2		1.062	0.166	0.036	0.015	0.023
3	1.357	0.926	0.173	0.029	0.008	0.004
4		0.969	0.207	0.068	0.023	0.038
5		0.790	0.176	0.077	0.015	0.011
6	1.474	0.855	0.167	0.049	0.012	0.014
7		0.981	0.206	0.084	0.025	0.034
8		0.787	0.212	0.076	0.020	0.029
9	1.171	0.740	0.217	0.082	0.021	0.025
10		0.677	0.154	0.157	0.029	0.019
14		0.708	0.159	0.120	0.021	0.006
15	1.114	0.761	0.191	0.157	0.029	0.010
16		0.704	0.166	0.147	0.027	0.017
17		0.607	0.225	0.167	0.031	0.023
18	1.229	0.543	0.209	0.217	0.035	0.007
19		0.611	0.197	0.193	0.038	0.033
20		0.594	0.187	0.217	0.038	0.024
21	1.292	0.545	0.156	0.217	0.037	0.013
22		0.586	0.212	0.213	0.039	0.022
23		0.511	0.203	0.185	0.031	0.018
24	0.792	0.541	0.239	0.230	0.042	0.025

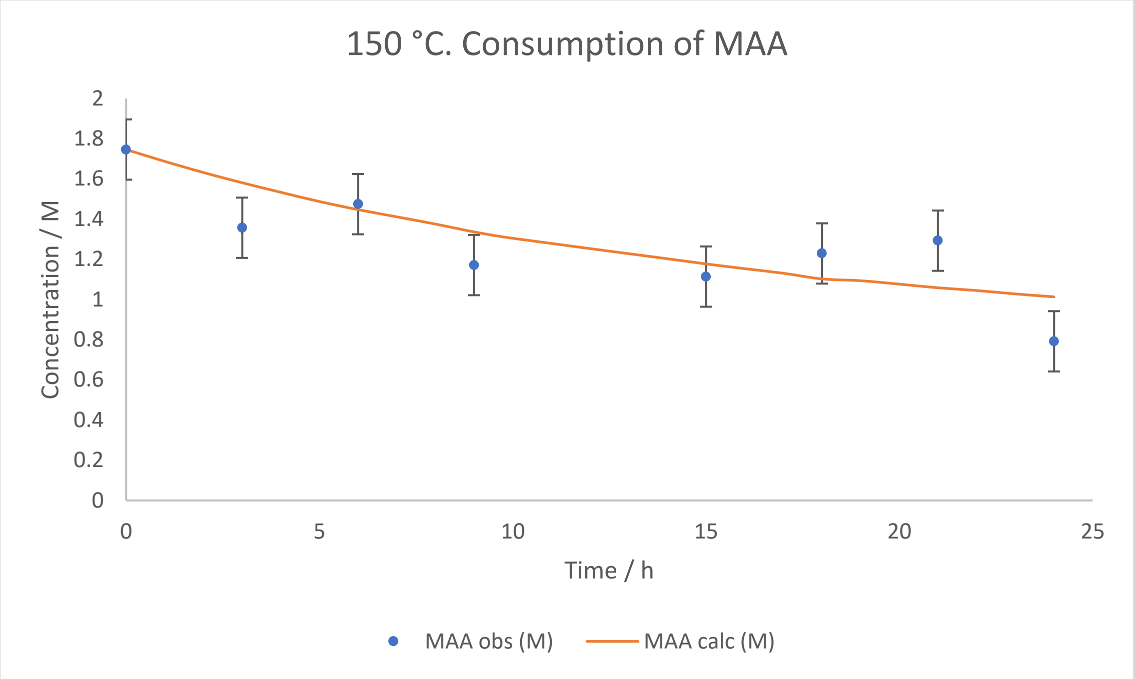

Figure S2. [**MAA**] versus time fitted to a 2nd order near equal concentrations regime at 150 °C, error bars represent 0.15 M.

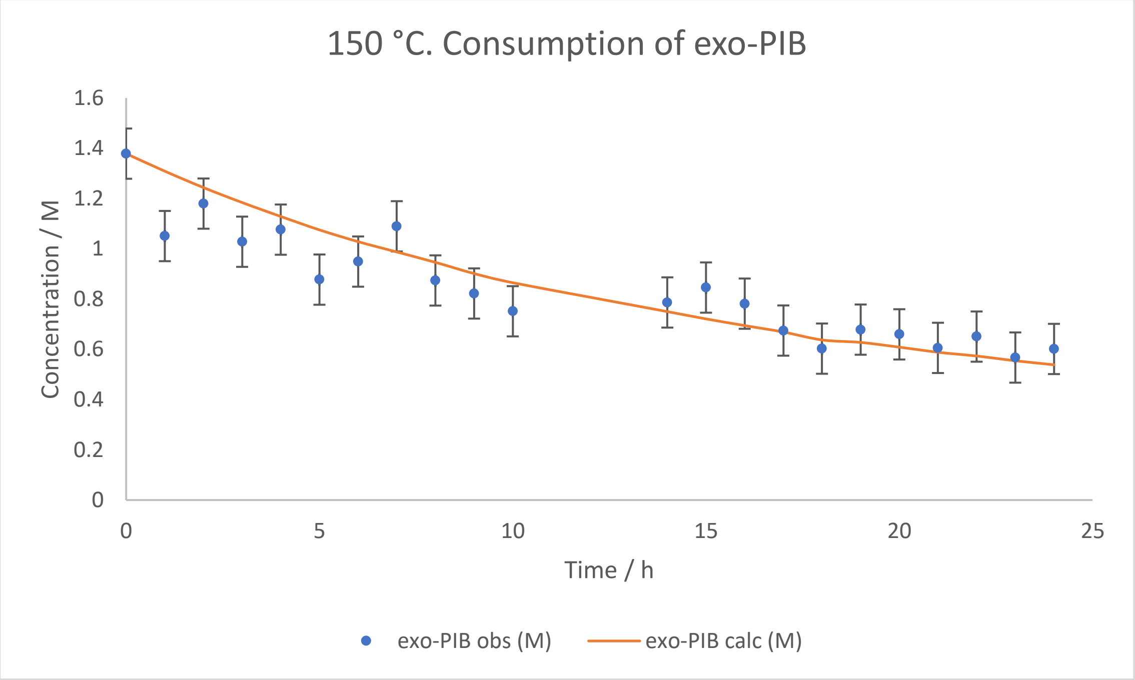

Figure S3. [*exo*-**PIB**] versus time fitted to a 2nd order near equal concentrations regime at 150 °C, error bars represent 0.1 M.

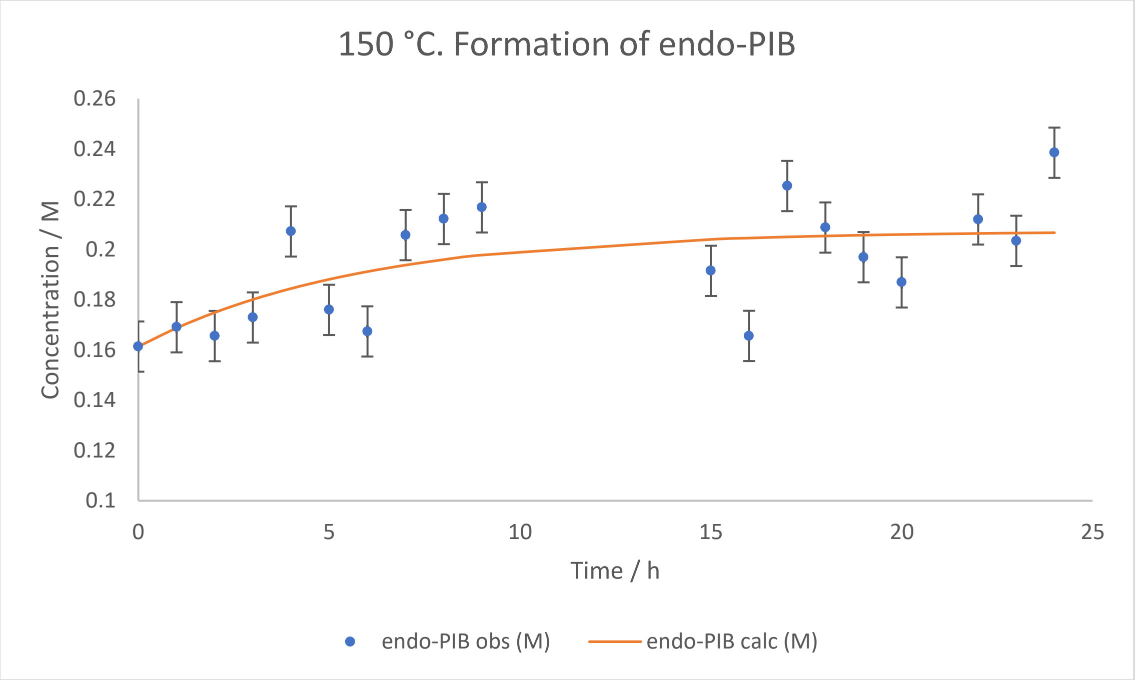

Figure S4. [*endo*-**PIB**] versus time fitted to a 1st order regime at 150 °C, error bars represent 0.01 M.

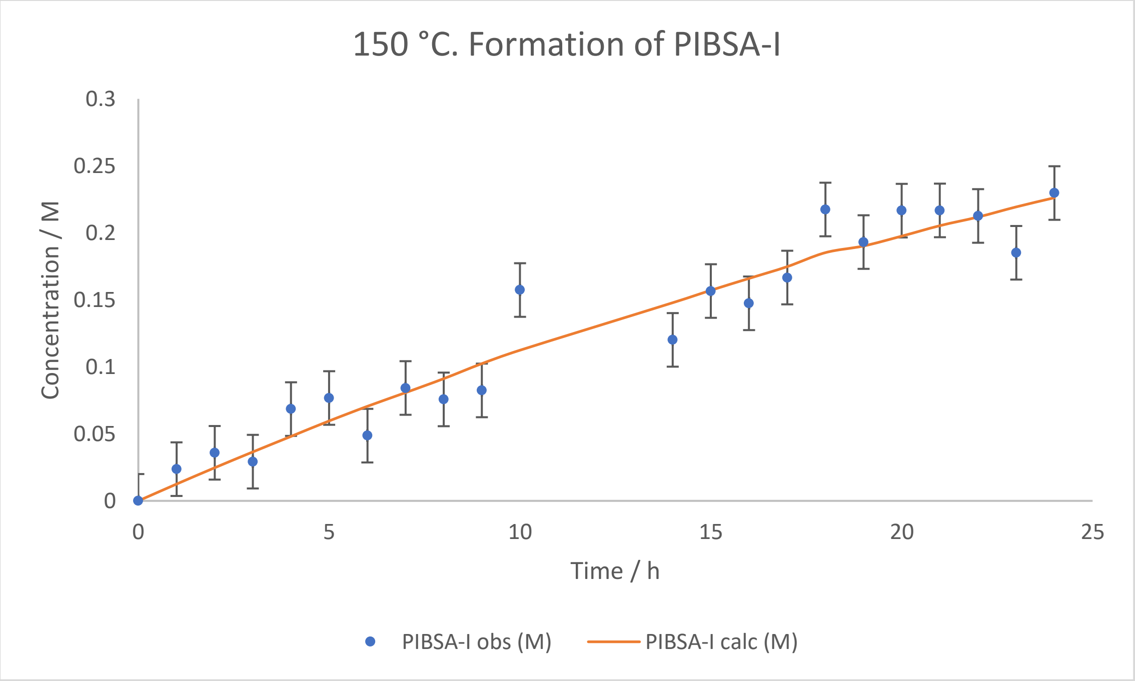

Figure S5. [**PIBSA-I**] versus time fitted to a 2nd order near equal concentrations regime at 150 °C, error bars represent 0.02 M.

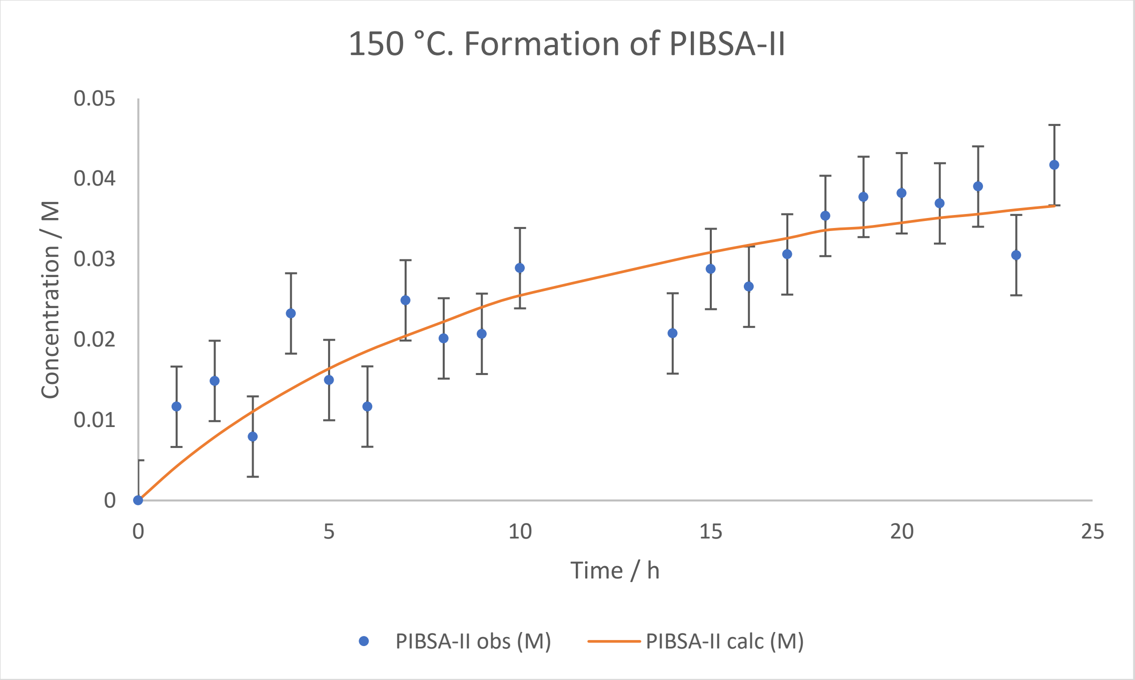

Figure S6. [**PIBSA-II**] versus time fitted to a 2nd order near equal concentrations regime at 150 °C, error bars represent 0.005 M.

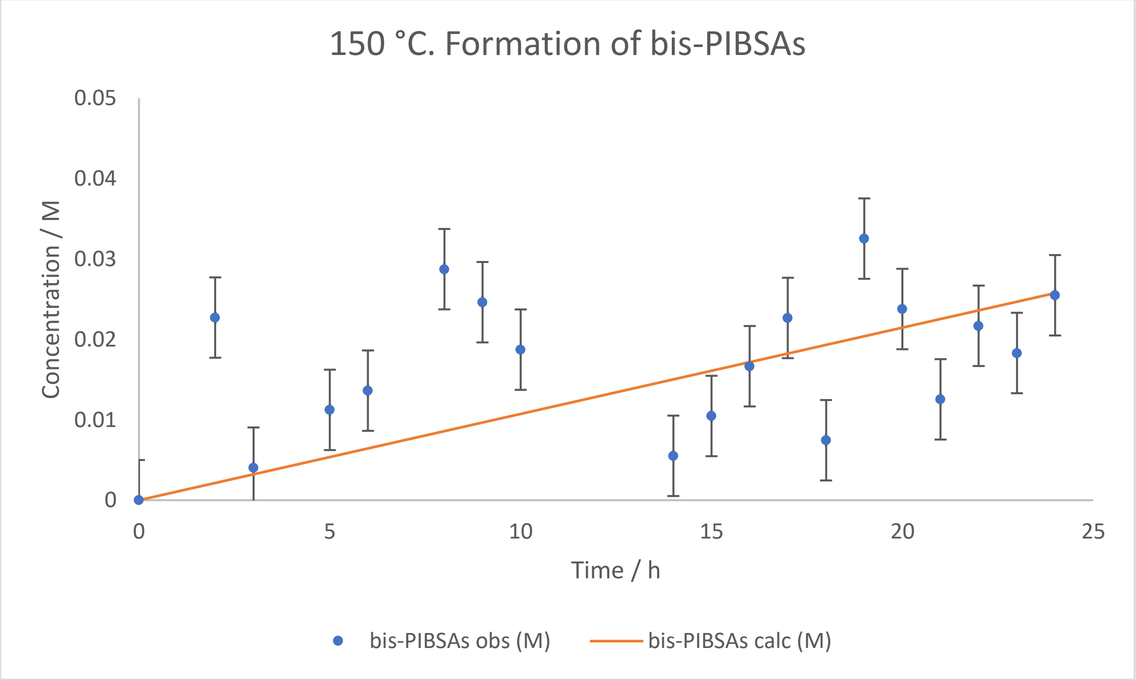

Figure S7. [*bis*-**PIBSAs**] versus time fitted to a 0th order regime at 150 °C. Only traces of this compound are realised at 150 °C leading to large scatter on the data. Error bars represent 0.005 M.

Table S3. Rate constant data at 150 °C.[a]

Species	k_{obs}[b]	k_1 (s^{-1})	R^2
Consumption of **MAA**	8(3) x 10^{-6}	1.0(4) x 10^{-5}	0.71
Consumption of *exo*-**PIB**	3.9(6) x 10^{-6}	7(1) x 10^{-6}	0.77
Formation of *endo*-**PIB**	5(3) x 10^{-5}	5(3) x 10^{-5}	0.39
Formation of **PIBSA-I**	3(2) x 10^{-6}	5(3) x 10^{-6}	0.93

Formation of **PIBSA-II**	1.6(6) x 10^{-5}	3(1) x 10^{-5}	0.84
Formation of *bis*-**PIBSAs**[c]	~1 x 10^{-3}	~6 x 10^{-4}	0.35

[a] Figures in parentheses indicate the estimated standard deviation in the last significant figure.

[b] Units of k_{obs} for **MAA**, *exo*-**PIB**, **PIBSA-I**, and **PIBSA-II** = M^{-1} s^{-1}. Units of k_{obs} for *endo*-**PIB** = s^{-1}. Units of k_{obs} for *bis*-**PIBSAs** = M s^{-1}.

[c] Only obtained in poor fit with large error due to low concentration.

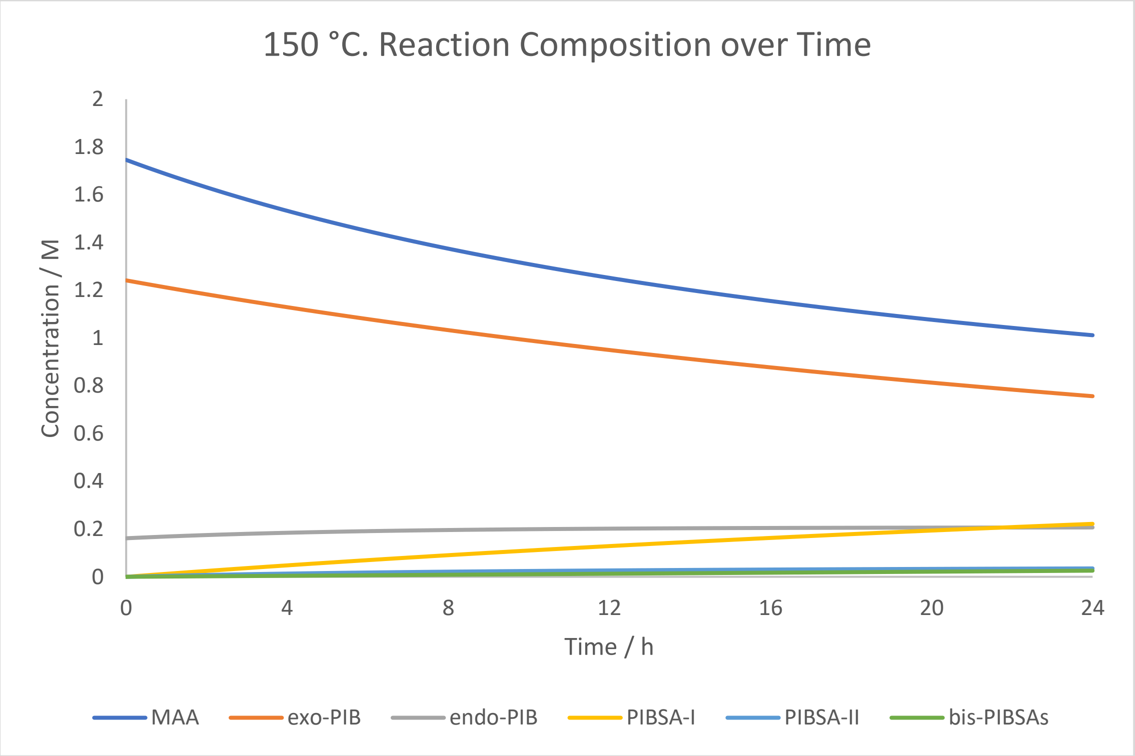

Figure S8. Simulated molarity of species over time at 150 °C using the data of Table S3.

Table S4. Reaction at 160 °C.

Time / h	**MAA** conc. (M)	*exo*-**PIB** conc. (M)	*endo*-**PIB** conc. (M)	**PIBSA-I** conc. (M)	**PIBSA-II** conc. (M)	*bis*-**PIBSAs** total conc. (M)
0	1.746	0.993	0.161	0.000	0.000	0.000
1		0.770	0.122	0.092	0.019	0.013
2		0.728	0.167	0.124	0.029	0.019
3	1.362	0.794	0.168	0.066	0.015	0.015
4		0.736	0.197	0.115	0.021	0.016
5		0.667	0.131	0.099	0.020	0.013
6	0.741	0.790	0.168	0.116	0.022	0.013
7		0.602	0.228	0.177	0.031	0.018
8		0.671	0.182	0.111	0.022	0.017
9	1.033	0.490	0.175	0.239	0.043	0.026
10		0.490	0.157	0.276	0.049	0.021
14		0.552	0.170	0.199	0.041	0.029
15	0.662	0.079	0.167	0.429	0.081	0.048
16		0.088	0.180	0.420	0.079	0.047
17		0.297	0.182	0.347	0.056	0.026
18	0.569	0.232	0.175	0.376	0.063	0.030

19		0.062	0.172	0.466	0.092	0.057
20		0.051	0.186	0.457	0.101	0.076
21	0.801	0.214	0.207	0.389	0.064	0.034
22		0.319	0.165	0.303	0.059	0.043
23		0.229	0.168	0.420	0.068	0.032
24	0.612	0.198	0.182	0.353	0.064	0.036

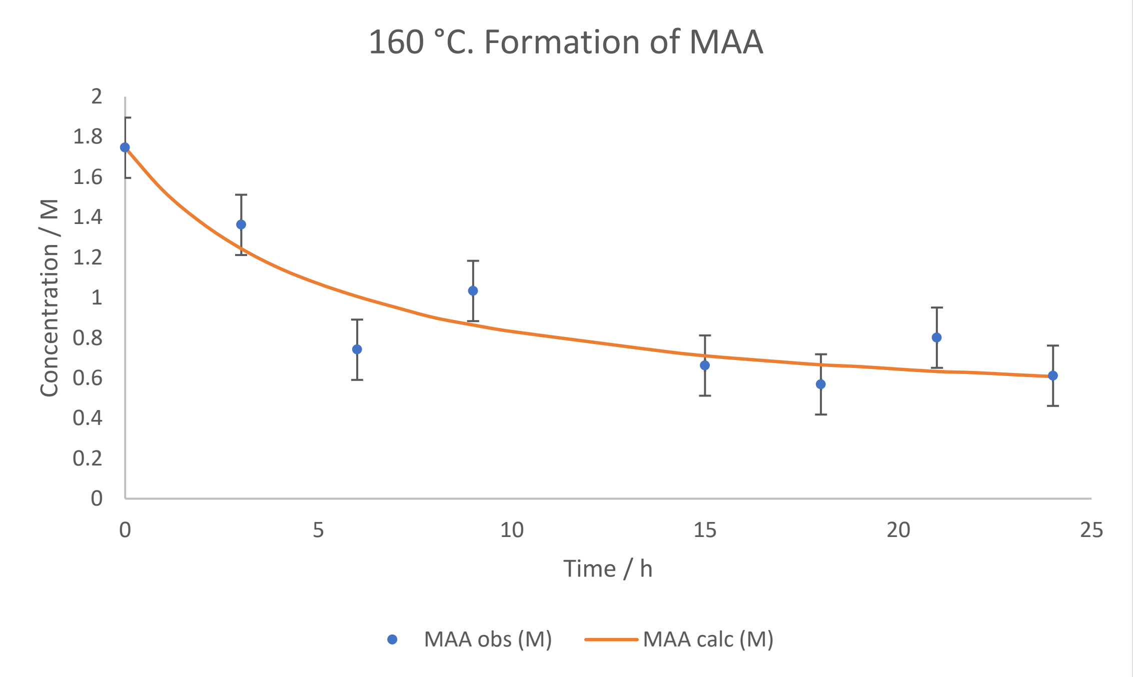

Figure S9. [**MAA**] versus time fitted to a 2nd order near equal concentrations regime at 160 °C, error bars represent 0.15 M.

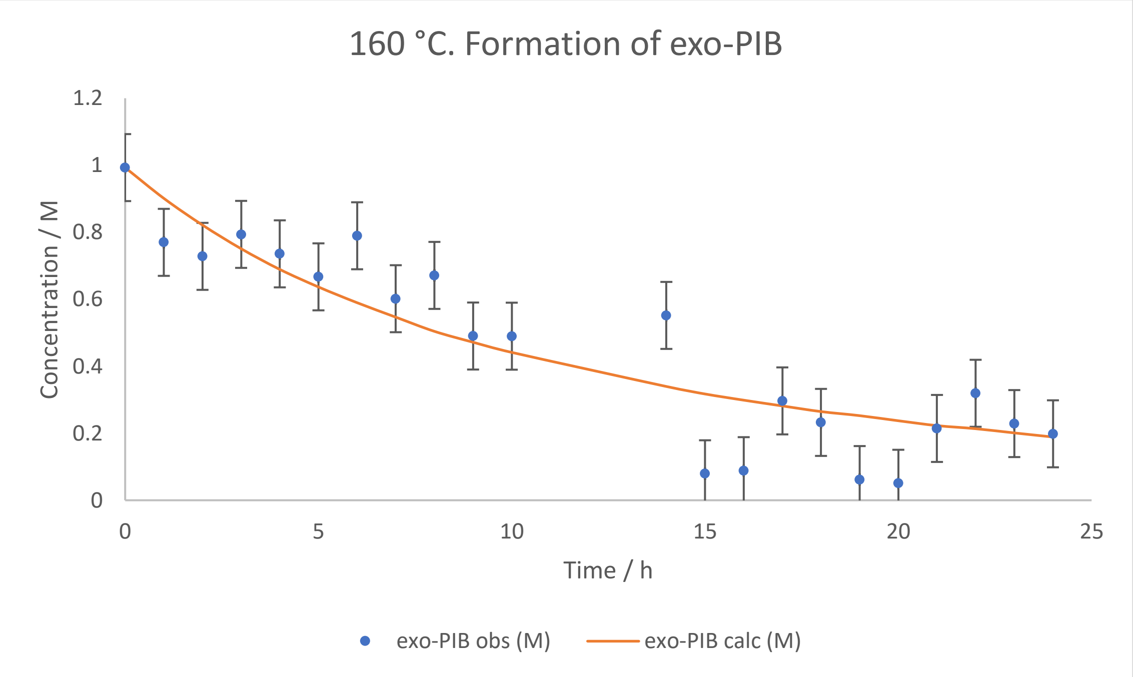

Figure S10. [exo-**PIB**] versus time fitted to a 2nd order near equal concentrations regime at 160 °C, error bars represent 0.1 M.

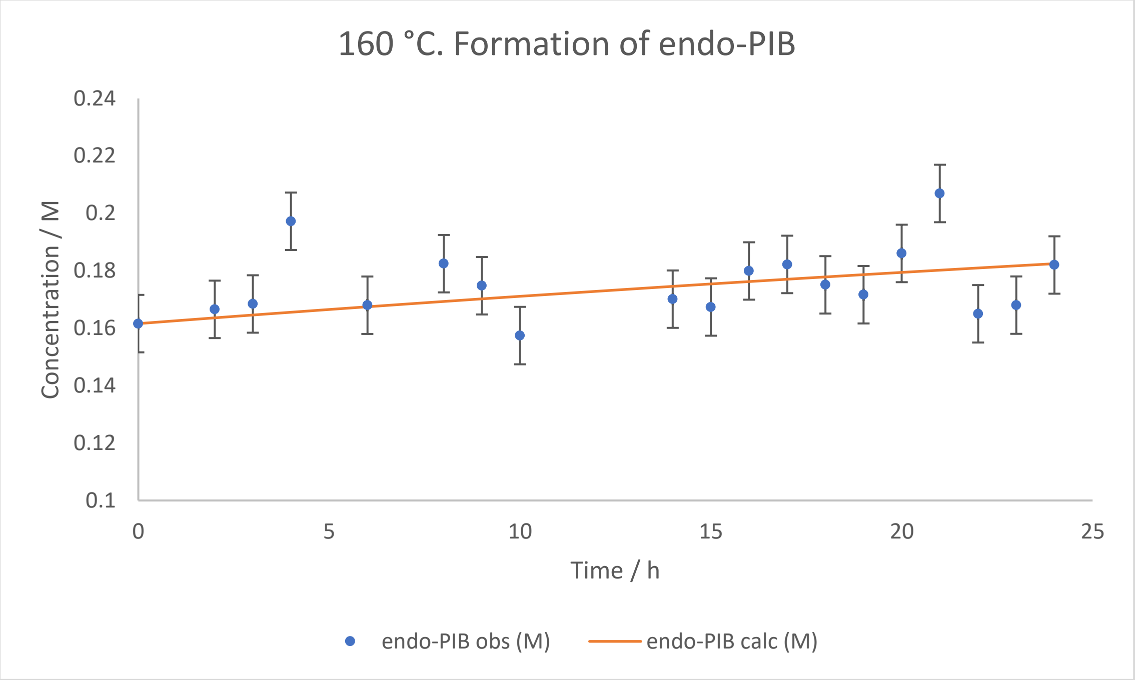

Figure S11. [*endo*-**PIB**] versus time fitted to 1st order regime at 160 °C, error bars represent 0.01 M.

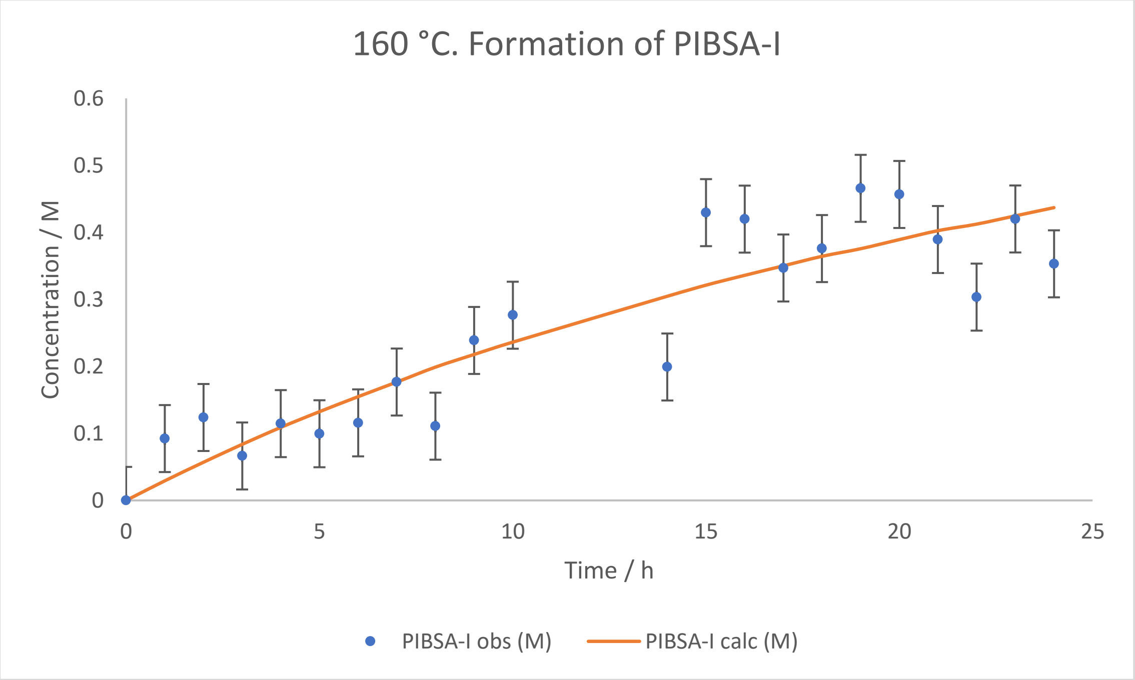

Figure S12. [**PIBSA-I**] versus time fitted to 2nd order near equal concentrations regime at 160 °C, error bars represent 0.05 M.

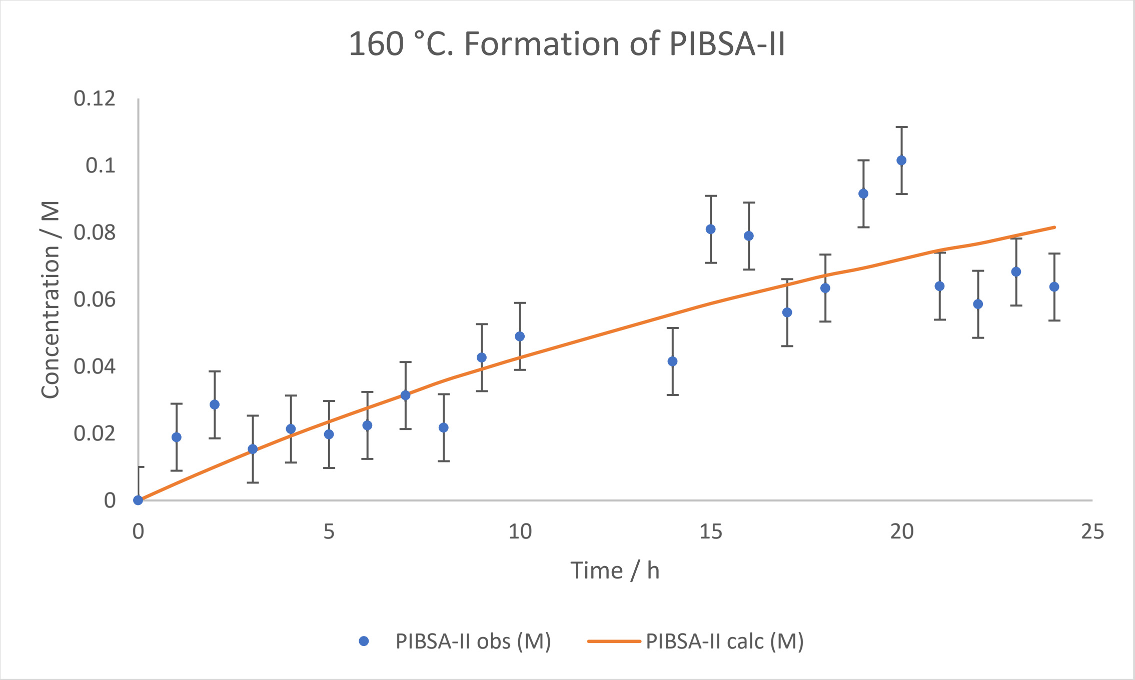

Figure S13. [**PIBSA-II**] versus time fitted to a 2nd order near equal concentrations regime at 160 °C, error bars represent 0.01 M.

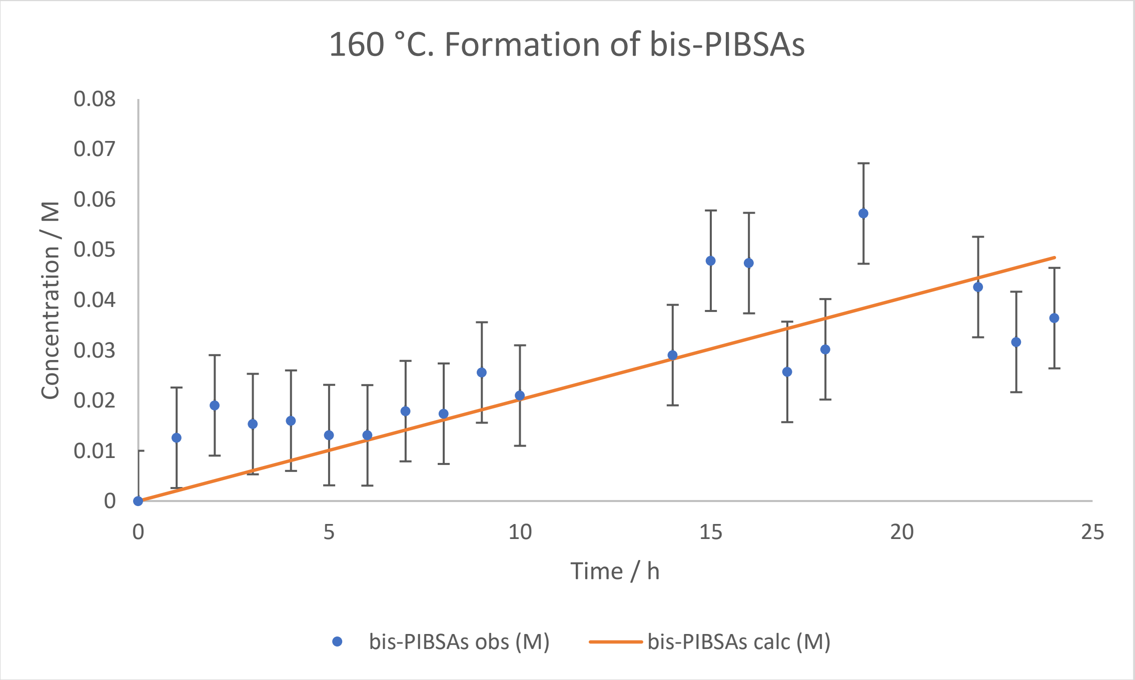

Figure S14. [*bis*-**PIBSAs**] versus time fitted to a 0th order regime at 160 °C, error bars represent 0.01 M.

Table S5. Rate constant data at 160 °C.[a]

Species	k_{obs}[b]	k_1 (s^{-1})	R^2
Consumption of **MAA**	3(1) x 10^{-5}	4(1) x 10^{-5}	0.88
Consumption of *exo*-**PIB**	1.6(3) x 10^{-5}	2.8(4) x 10^{-5}	0.79
Formation of *endo*-**PIB**	4(3) x 10^{-6}	4(3) x 10^{-6}	0.24

Formation of **PIBSA-I**	5(4) x 10^{-6}	9(6) x 10^{-6}	0.83
Formation of **PIBSA-II**	5(4) x 10^{-6}	8(7) x 10^{-6}	0.78
Formation of *bis*-**PIBSAs**[c]	~2 x 10^{-3}	~1 x 10^{-3}	0.72

[a] Figures in parentheses indicate the estimated standard deviation in the last significant figure.

[b] Units of k_{obs} for **MAA**, *exo*-**PIB**, **PIBSA-I**, and **PIBSA-II** = M^{-1} s^{-1}. Units of k_{obs} for *endo*-**PIB** = s^{-1}. Units of k_{obs} for *bis*-**PIBSA** = M s^{-1}.

[c] Only obtained in poor fit with large error due to low concentration.

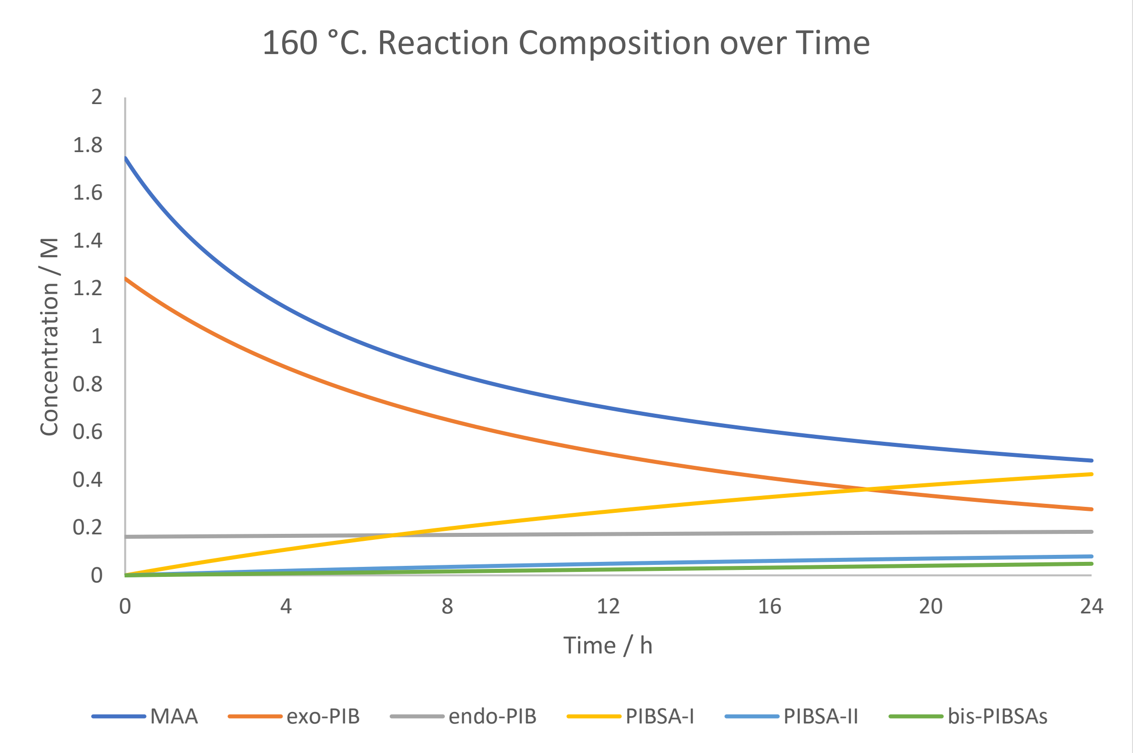

Figure S15. Simulated molarity of species over time at 160 °C using the data from Table S5.

Table S6. Reaction at 165 °C.

Time / h	**MAA** conc. (M)	*exo*-**PIB** conc. (M)	*endo*-**PIB** conc. (M)	**PIBSA-I** conc. (M)	**PIBSA-II** conc. (M)	*bis*-**PIBSAs** total conc. (M)
0	1.746	0.993	0.161	0.000	0.000	0.000
1		1.029	0.153	0.032	0.013	0.020
2		0.880	0.189	0.056	0.013	0.013
3	1.230	0.789	0.164	0.130	0.024	0.015
4		0.666	0.243	0.157	0.031	0.019
5		0.698	0.156	0.139	0.029	0.017
6	1.377	0.568	0.187	0.169	0.033	0.024
7		0.418	0.200	0.261	0.047	0.024
8		0.658	0.196	0.169	0.033	0.021
9	1.146	0.408	0.216	0.292	0.049	0.018
10		0.332	0.180	0.315	0.054	0.023
14		0.241	0.179	0.384	0.066	0.027
15	0.693	0.244	0.193	0.407	0.069	0.034

16		0.403	0.151	0.254	0.053	0.039
17		0.256	0.185	0.332	0.063	0.039
18		0.223	0.192	0.396	0.071	0.036
19	0.886	0.344	0.186	0.265	0.055	0.043
20		0.162	0.205	0.416	0.076	0.043
21	0.754	0.137	0.232	0.423	0.071	0.039
22		0.087	0.194	0.443	0.079	0.053
23		0.251	0.201	0.330	0.063	0.041
24	0.309	0.147	0.205	0.385	0.072	0.050

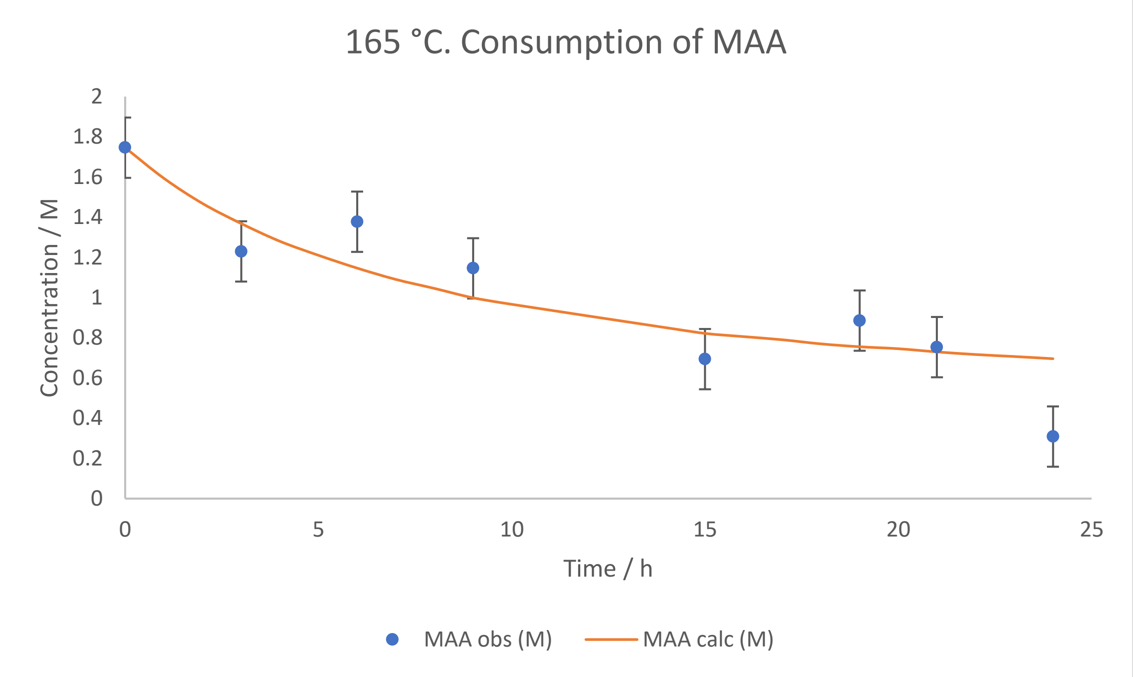

Figure S16. [**MAA**] versus time fitted to a 2nd order near equal concentrations regime at 165 °C, error bars represent 0.15 M.

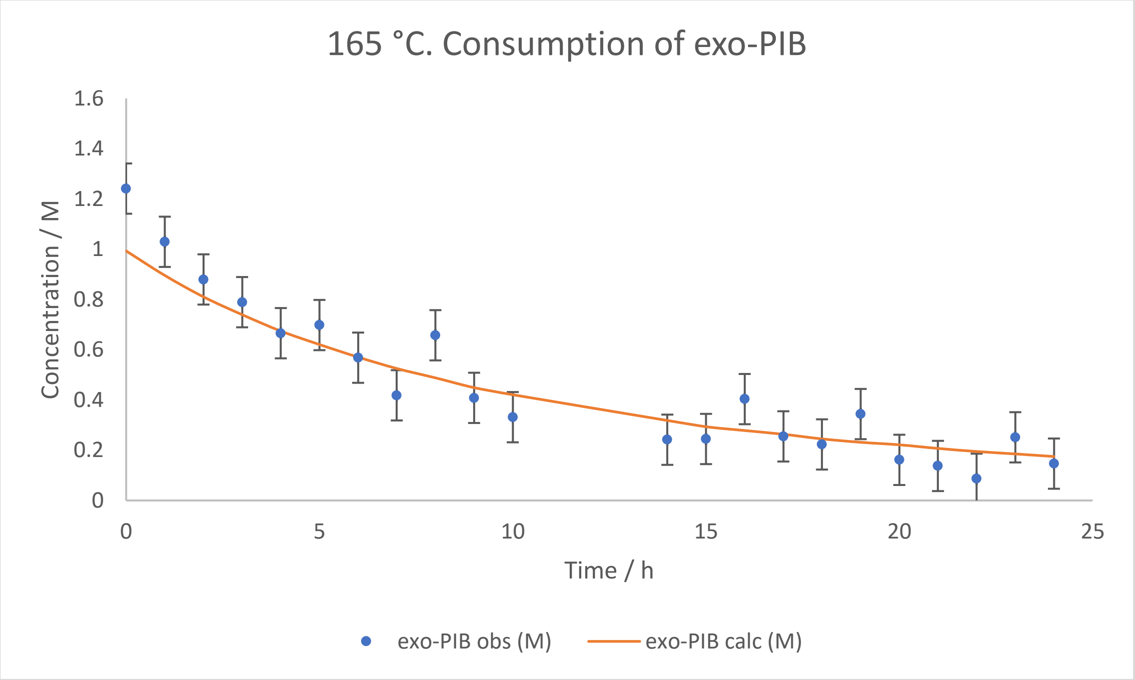

Figure S17. [*exo*-**PIB**] versus time fitted to a 2nd order near equal concentrations regime at 165 °C, error bars represent 0.1 M.

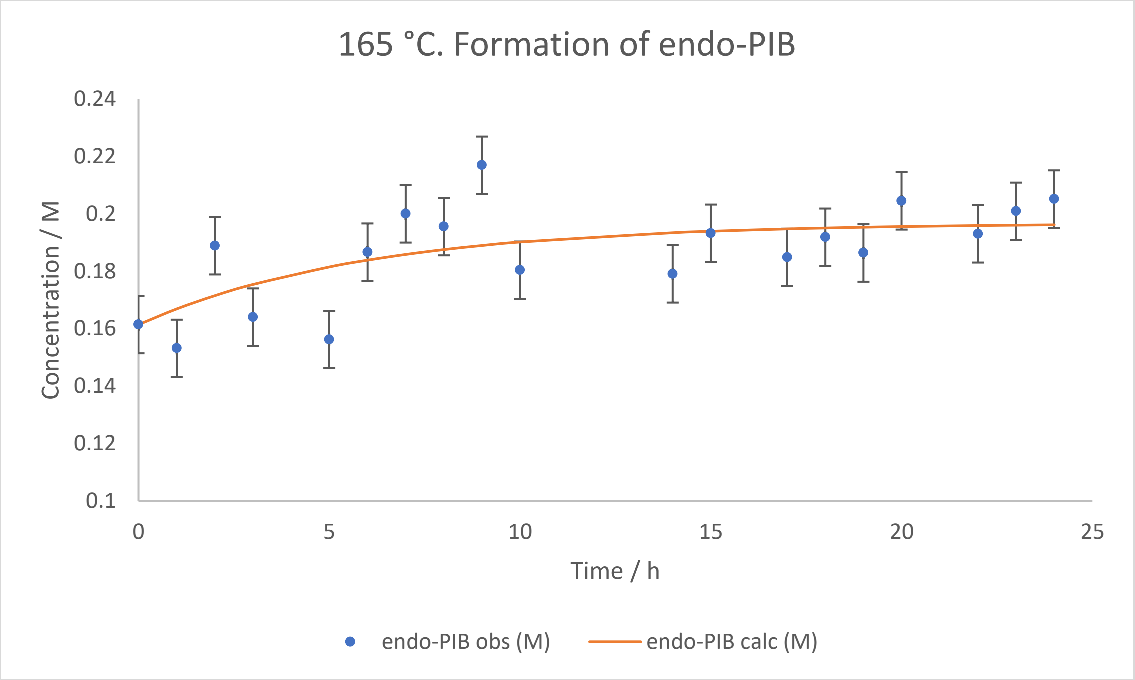

Figure S18. [*endo*-**PIB**] versus time fitted to a 1st order regime at 165 °C, error bars represent 0.01 M.

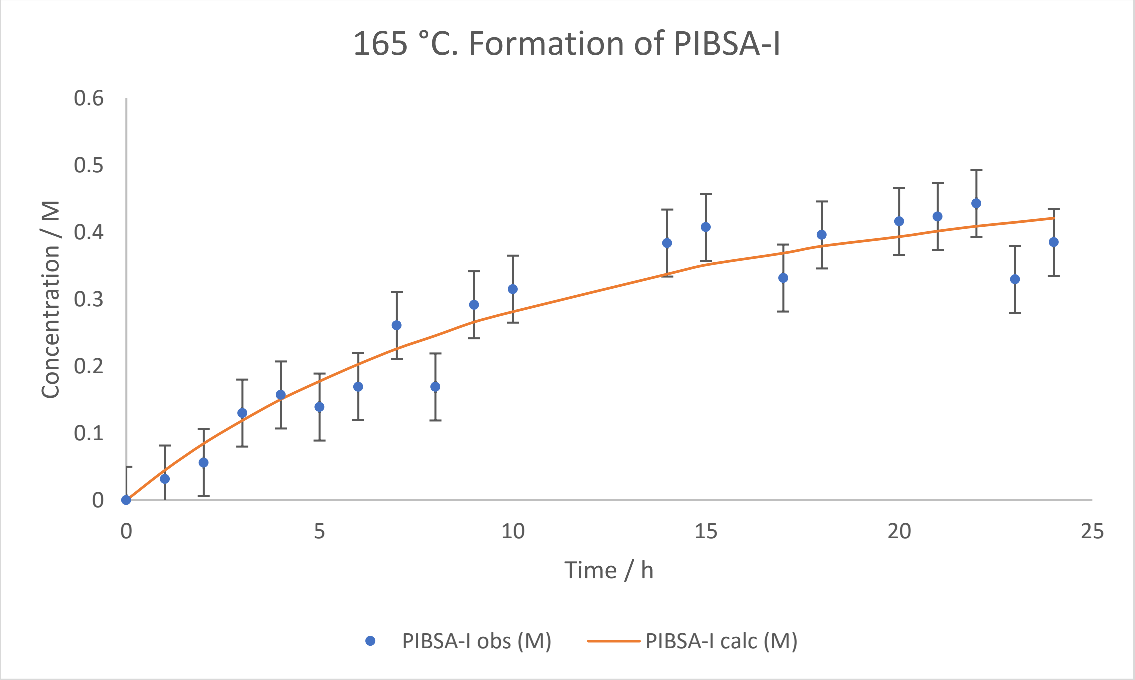

Figure S19. [**PIBSA-I**] versus time fitted to a 2nd order near equal concentrations regime at 165 °C, error bars represent 0.05 M.

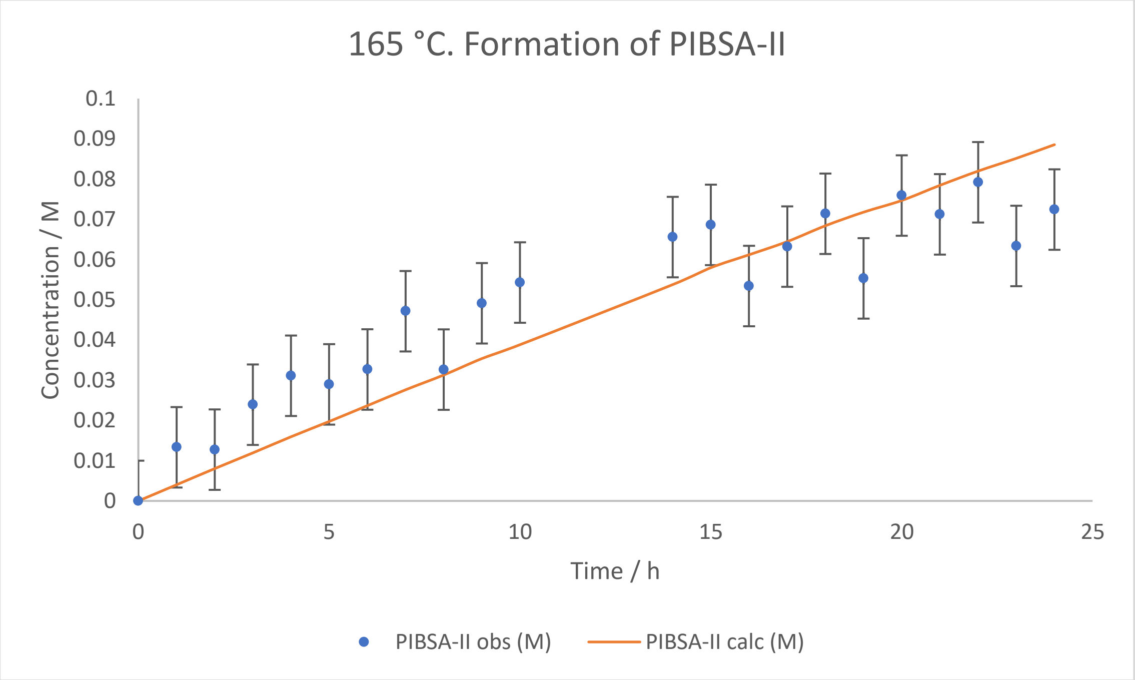

Figure S20. [**PIBSA-II**] versus time fitted to a 2nd order near equal concentrations regime at 165 °C, error bars represent 0.01 M.

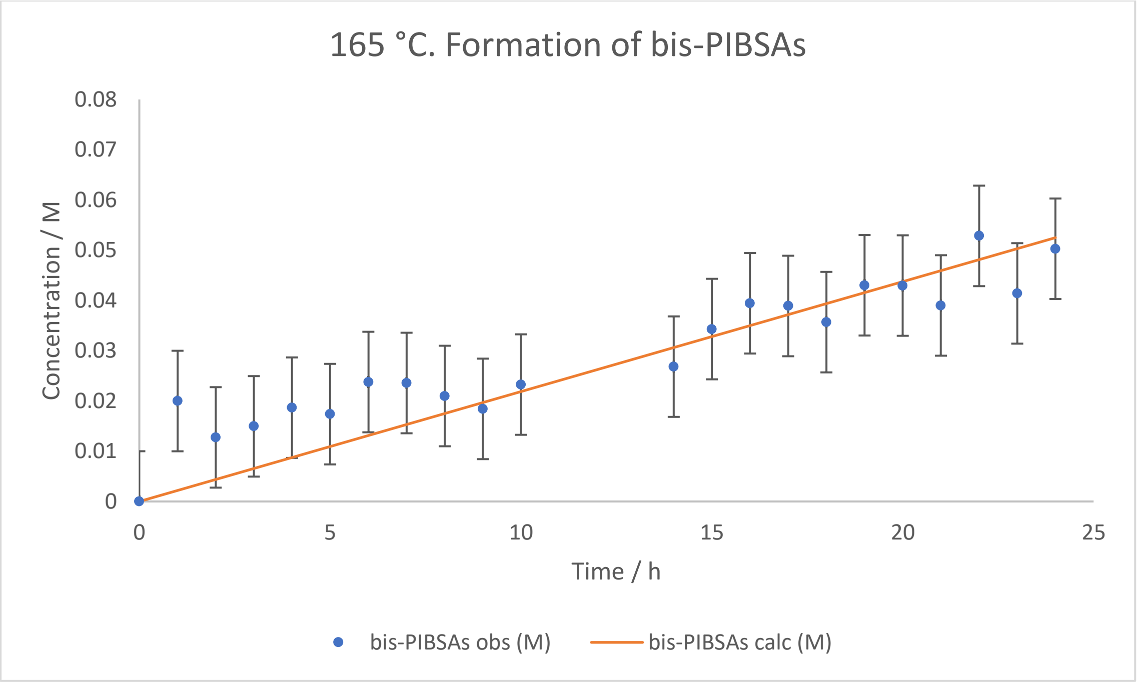

Figure S21. [*bis*-**PIBSAs**] versus time fitted to a 0th order regime at 165 °C, error bars represent 0.01 M.

Table S7. Rate constant data at 165 °C.[a]

Species	k_{obs}[b]	k_1 (s^{-1})	R^2
Consumption of **MAA**	2(1) x10^{-5}	2(1) x10^{-5}	0.78
Consumption of *exo*-**PIB**	1.7(2) x10^{-5}	3.0(4) x10^{-6}	0.87
Formation of *endo*-**PIB**	5(3) x10^{-5}	5(3) x10^{-5}	0.41
Formation of **PIBSA-I**	1.4(4) x10^{-5}	2.5(6) x10^{-5}	0.92
Formation of **PIBSA-II**	1(2) x10^{-6}	1(4) x10^{-6}	0.86
Formation of *bis*-**PIBSAs**[c]	~2 x10^{-3}	~1 x10^{-3}	0.96

[a] Figures in parentheses indicate the estimated standard deviation in the last significant figure.

[b] Units of k_{obs} for **MAA**, *exo*-**PIB**, **PIBSA-I**, and **PIBSA-II** = M^{-1} s^{-1}. Units of k_{obs} for *endo*-**PIB** = s^{-1}. Units of k_{obs} for *bis*-**PIBSAs** = M s^{-1}.

[c] Only obtained in poor fit with large error due to low concentration.

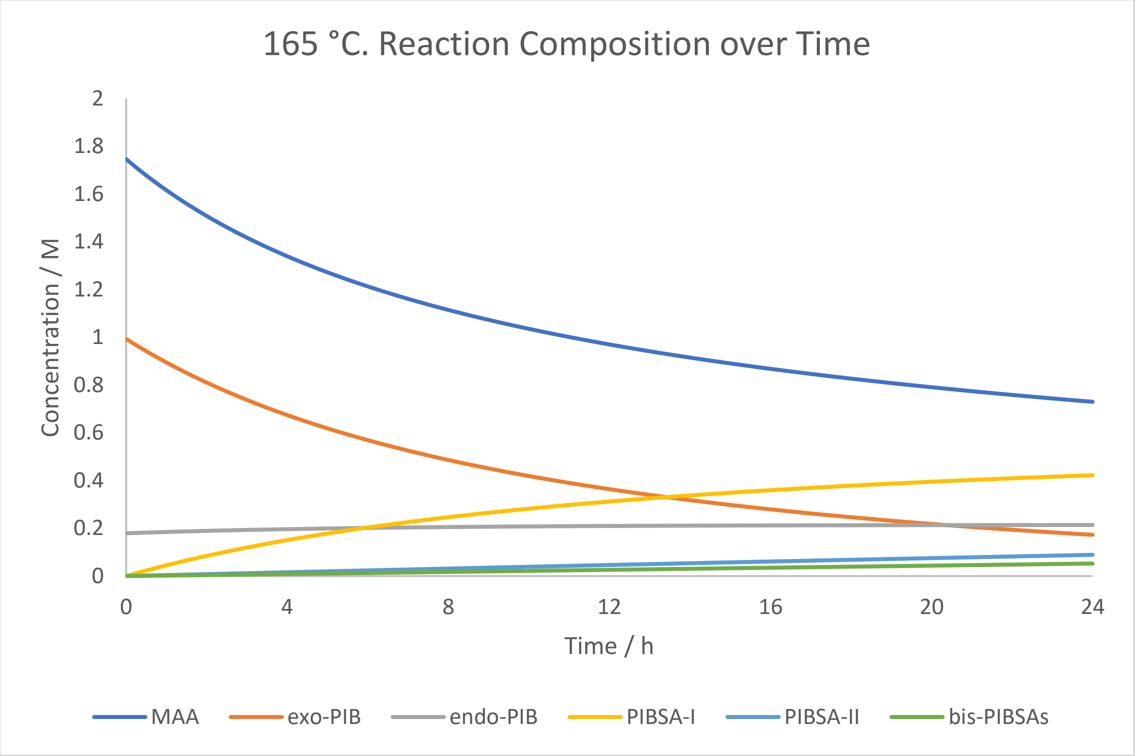

Figure S22. Simulated molarity of species over time at 165 °C using the data from Table S7.

Table S8. Reaction at 170 °C.

Time / h	**MAA** conc. (M)	*exo*-**PIB** conc. (M)	*endo*-**PIB** conc. (M)	**PIBSA-I** conc. (M)	**PIBSA-II** conc. (M)	*bis*-**PIBSAs** total conc. (M)
0	1.746	0.993	0.161	0.000	0.000	0.000
1		0.885	0.172	0.045	0.013	0.017
2		0.780	0.129	0.050	0.013	0.011
3	1.199	0.647	0.164	0.101	0.022	0.017
4		0.590	0.154	0.131	0.025	0.017
5		0.723	0.154	0.177	0.035	0.017
6	1.075	0.345	0.174	0.310	0.052	0.020
7		0.308	0.184	0.324	0.054	0.024
8		0.241	0.192	0.369	0.063	0.030
9		0.324	0.171	0.397	0.073	0.030
10	0.728	0.196	0.167	0.383	0.065	0.028
14		0.326	0.163	0.267	0.055	0.039
15	0.752	0.212	0.287	0.258	0.071	0.041
16		0.252	0.176	0.342	0.066	0.038
17	0.469	0.077	0.195	0.364	0.082	0.047
18		0.271	0.189	0.239	0.064	0.054
19		0.083	0.188	0.470	0.087	0.052
20		0.100	0.179	0.474	0.084	0.043
21	0.526	0.059	0.166	0.459	0.087	0.059
22		0.064	0.205	0.447	0.083	0.064
23		0.170	0.192	0.344	0.071	0.060
24	0.424	0.092	0.186	0.386	0.083	0.066

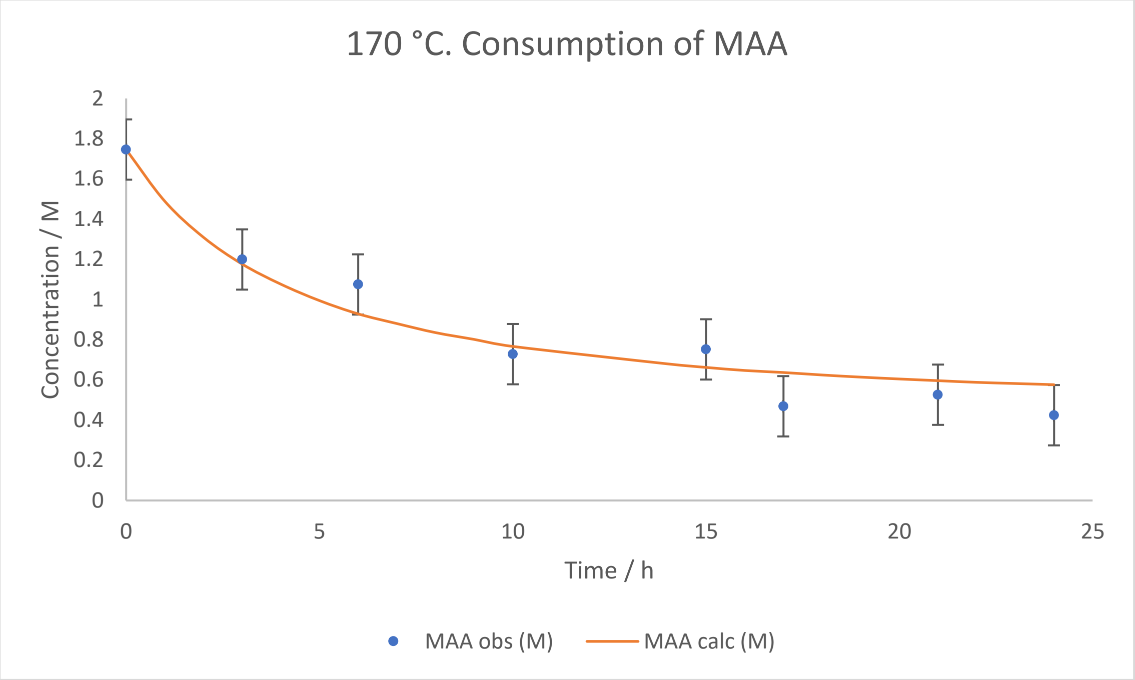

Figure S23. [**MAA**] versus time fitted to a 2nd order near equal concentrations regime at 170 °C, error bars represent 0.15 M.

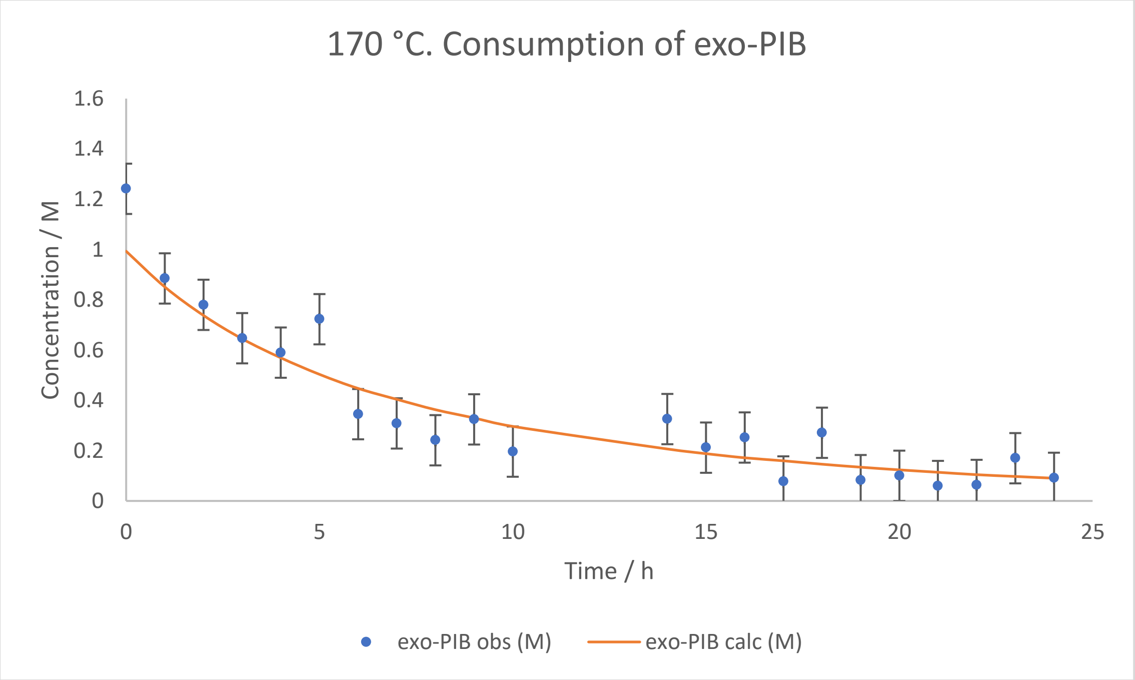

Figure S24. [*exo*-**PIB**] versus time fitted to a 2nd order near equal concentrations regime at 170 °C, error bars represent 0.1 M.

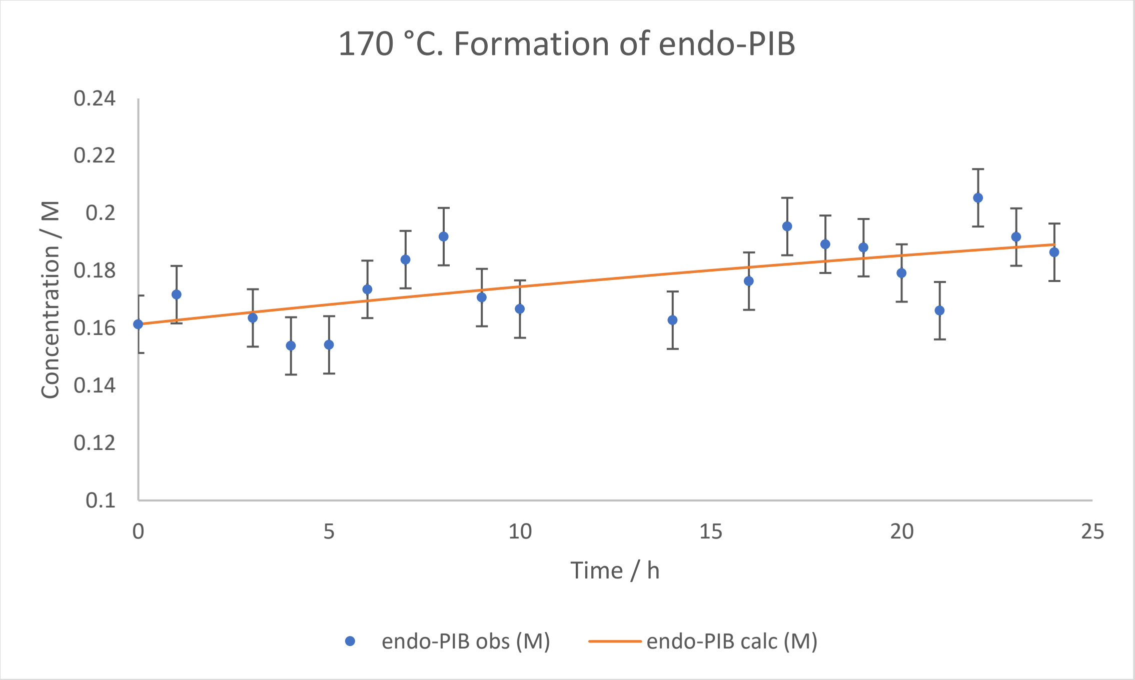

Figure S25. [*endo*-**PIB**] versus time fitted to a 1st order regime at 170 °C, error bars represent 0.01 M.

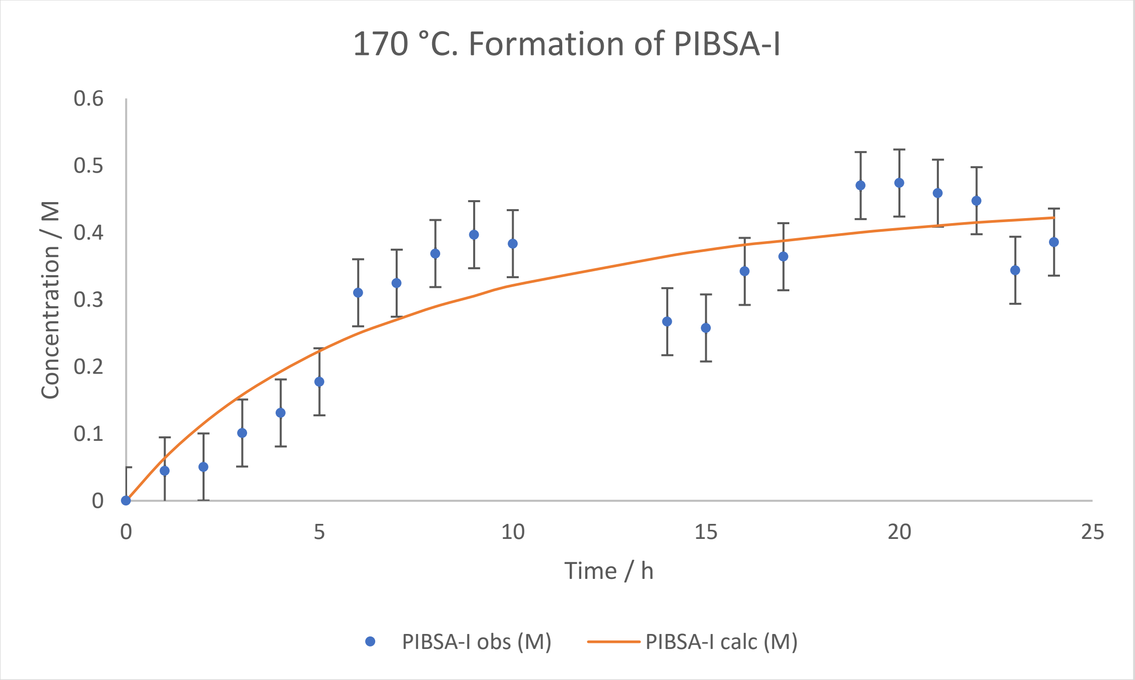

Figure S26. [**PIBSA-I**] versus time fitted to a 2nd order near equal concentrations regime at 170 °C, error bars represent 0.05 M.

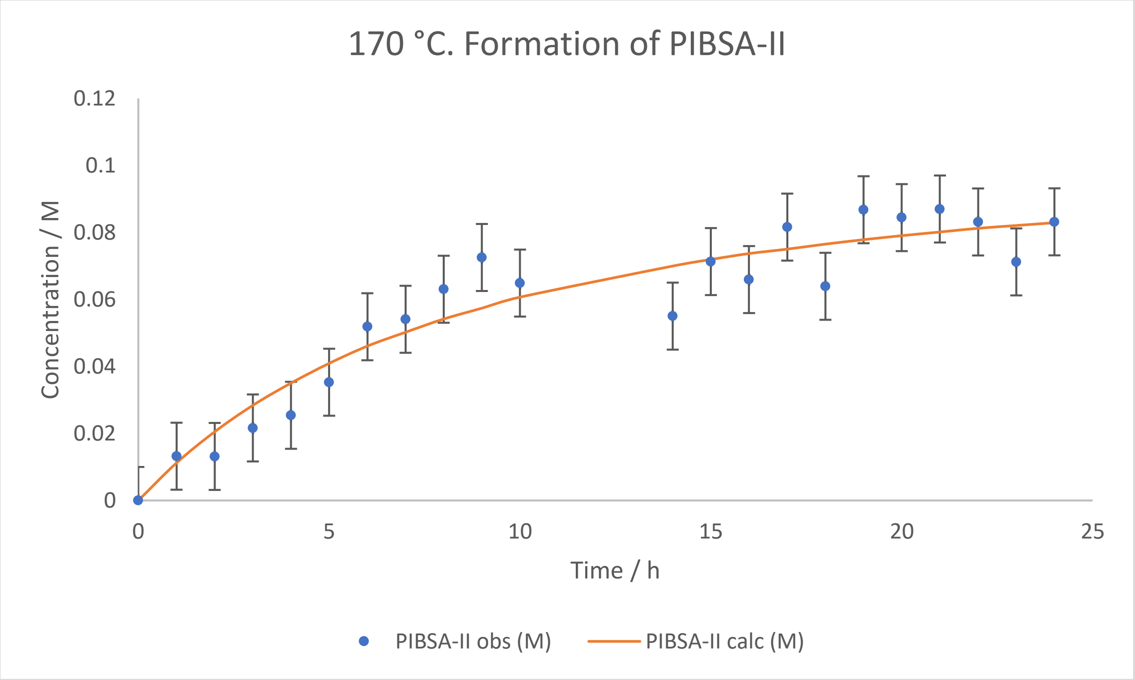

Figure S27. [**PIBSA-II**] versus time fitted to a 2nd order near equal concentrations regime at 170 °C, error bars represent 0.01 M.

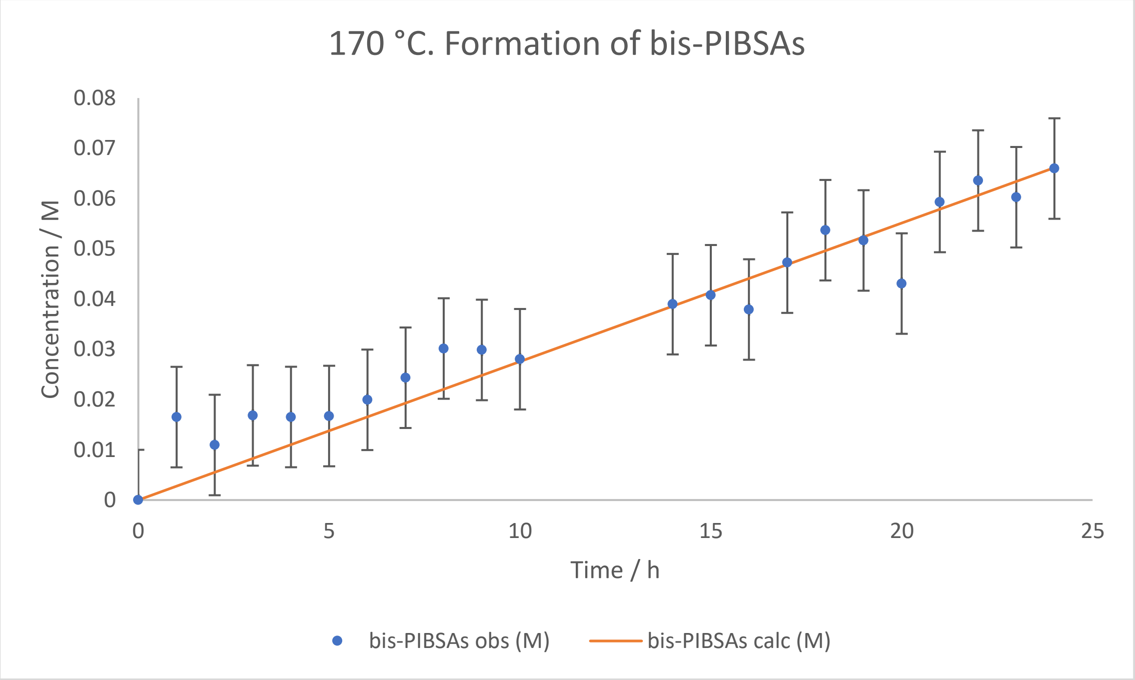

Figure S28. [*bis*-**PIBSAs**] versus time fitted to a 0th order regime at 170 °C, error bars represent 0.01 M.

Table S9. Rate constant data at 170 °C.[a]

Species	k_{obs}[b]	k_1 (s^{-1})	R^2
Consumption of **MAA**	4(1) x 10^{-5}	5(1) x 10^{-5}	0.93
Consumption of *exo*-**PIB**	2.6(3) x 10^{-5}	4.5(6) x 10^{-5}	0.87
Formation of *endo*-**PIB**	1(2) x 10^{-5}	1(2) x 10^{-5}	0.39

Formation of **PIBSA-I**	2.4(8) x 10^{-5}	4(1) x 10^{-5}	0.79
Formation of **PIBSA-II**	2.1(5) x 10^{-5}	3.6(8) x 10^{-5}	0.91
Formation of *bis*-**PIBSAs**[c]	~3 x 10^{-3}	~2 x 10^{-3}	0.94

[a] Figures in parentheses indicate the estimated standard deviation in the last significant figure.

[b] Units of k_{obs} for **MAA**, *exo*-**PIB**, **PIBSA-I**, and **PIBSA-II** = M^{-1} s^{-1}. Units of k_{obs} for *endo*-**PIB** = s^{-1}. Units of k_{obs} for *bis*-**PIBSAs** = M s^{-1}.

[c] Only obtained in poor fit with large error due to low concentration.

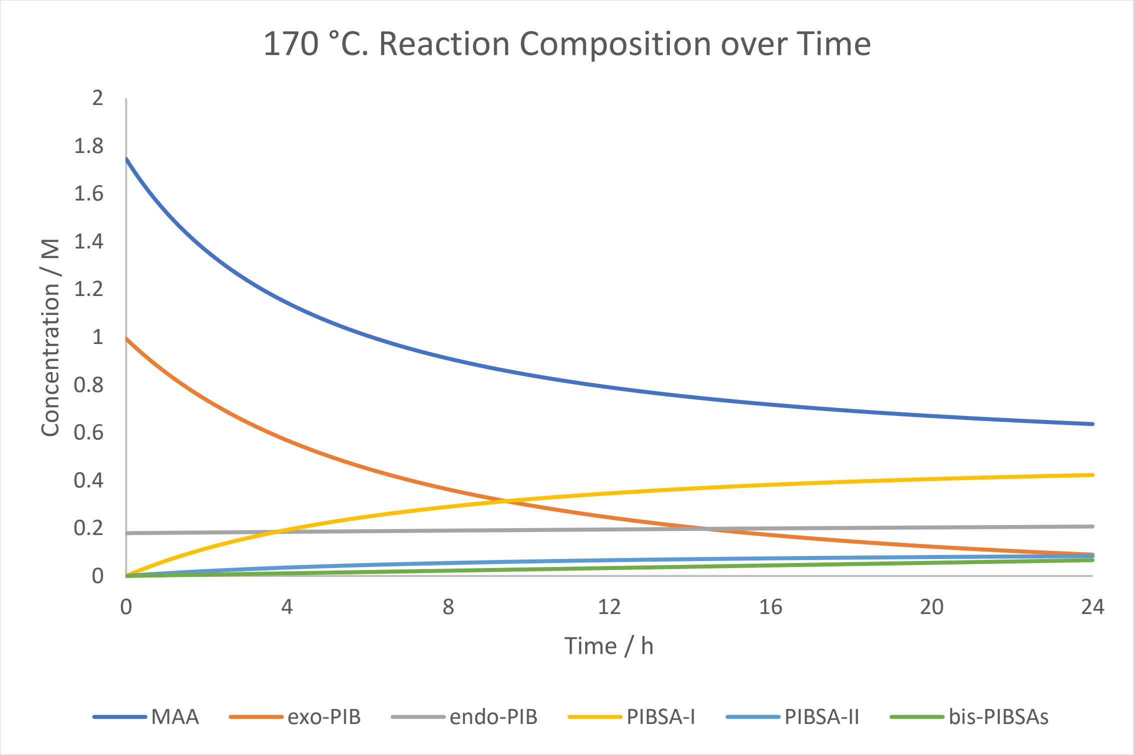

Figure S29. Simulated molarity of species over time at 170 °C using the data of Table S9.

Table S10. Reaction at 180 °C.

Time / h	**MAA** conc. (M)	*exo*-**PIB** conc. (M)	*endo*-**PIB** conc. (M)	**PIBSA-I** conc. (M)	**PIBSA-II** conc. (M)	*bis*-**PIBSAs** total conc. (M)
0	1.746	0.993	0.161	0.000	0.000	0.000
1		0.761	0.171	0.082	0.020	0.018
2		0.586	0.224	0.198	0.041	0.033
3	1.185	0.488	0.201	0.200	0.044	0.036
4		0.336	0.179	0.312	0.058	0.035
5		0.377	0.186	0.302	0.052	0.025
6	0.917	0.313	0.205	0.313	0.066	0.052
7		0.099	0.120	0.275	0.050	0.023
8		0.465	0.205	0.294	0.070	0.043
9	0.704	0.355	0.161	0.203	0.052	0.043
10		0.153	0.175	0.377	0.081	0.059
14		0.069	0.166	0.450	0.087	0.049
15	0.613	0.180	0.183	0.314	0.084	0.093

16		0.208	0.187	0.370	0.073	0.025
17		0.036	0.176	0.435	0.093	0.067
18	0.567	0.049	0.186	0.403	0.087	0.058
19		0.125	0.174	0.327	0.086	0.078
20		0.158	0.184	0.312	0.091	0.105
21	0.505	0.032	0.178	0.415	0.096	0.078
22		0.064	0.186	0.401	0.098	0.083
23		0.165	0.167	0.271	0.084	0.084
24	0.384	0.041	0.215	0.390	0.095	0.091

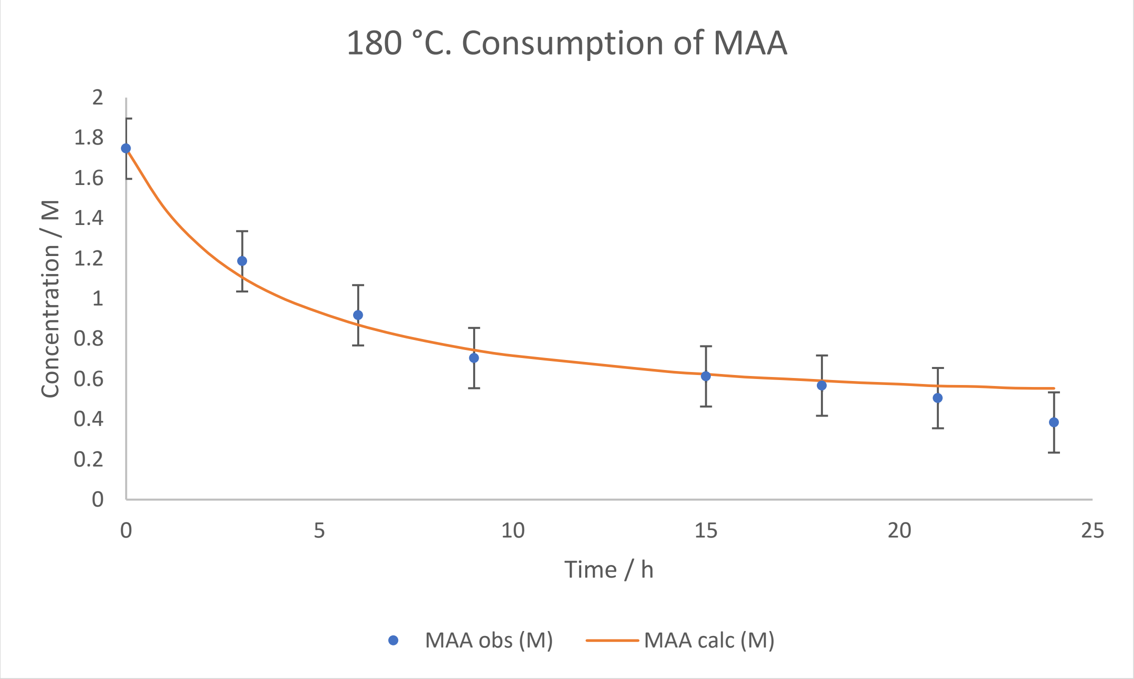

Figure S30. [**MAA**] versus time fitted to a 2nd order near equal concentrations regime at 180 °C, error bars represent 0.15 M.

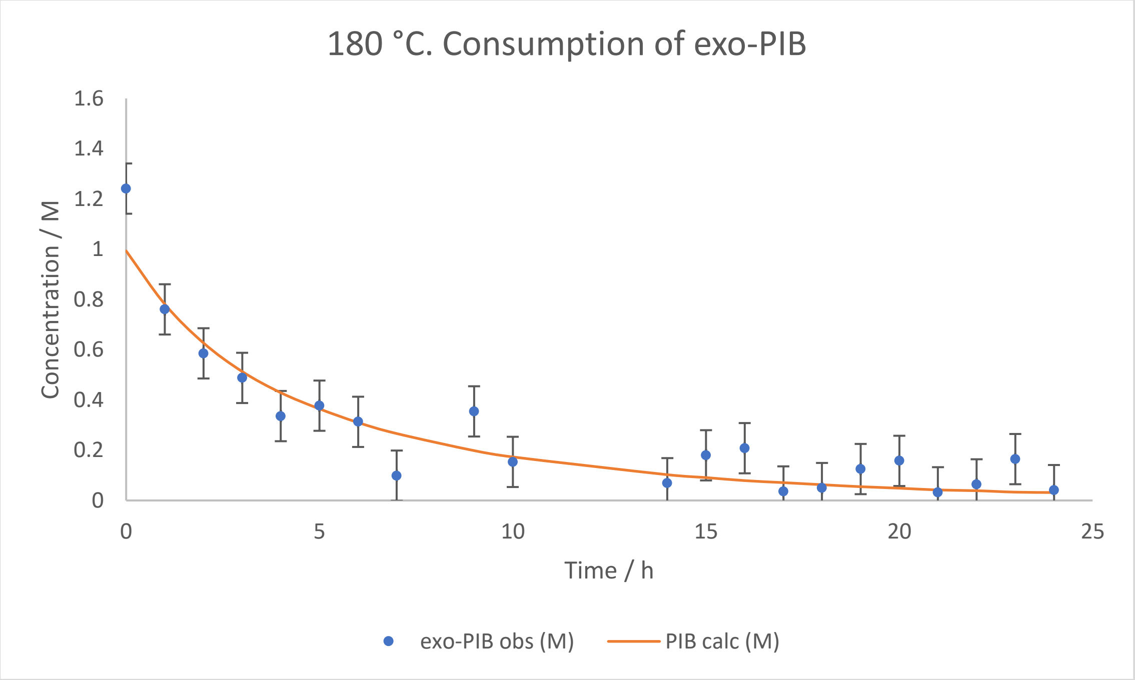

Figure S31. [*exo*-**PIB**] versus time fitted to a 2nd order near equal concentrations regime at 180 °C, error bars represent 0.1 M.

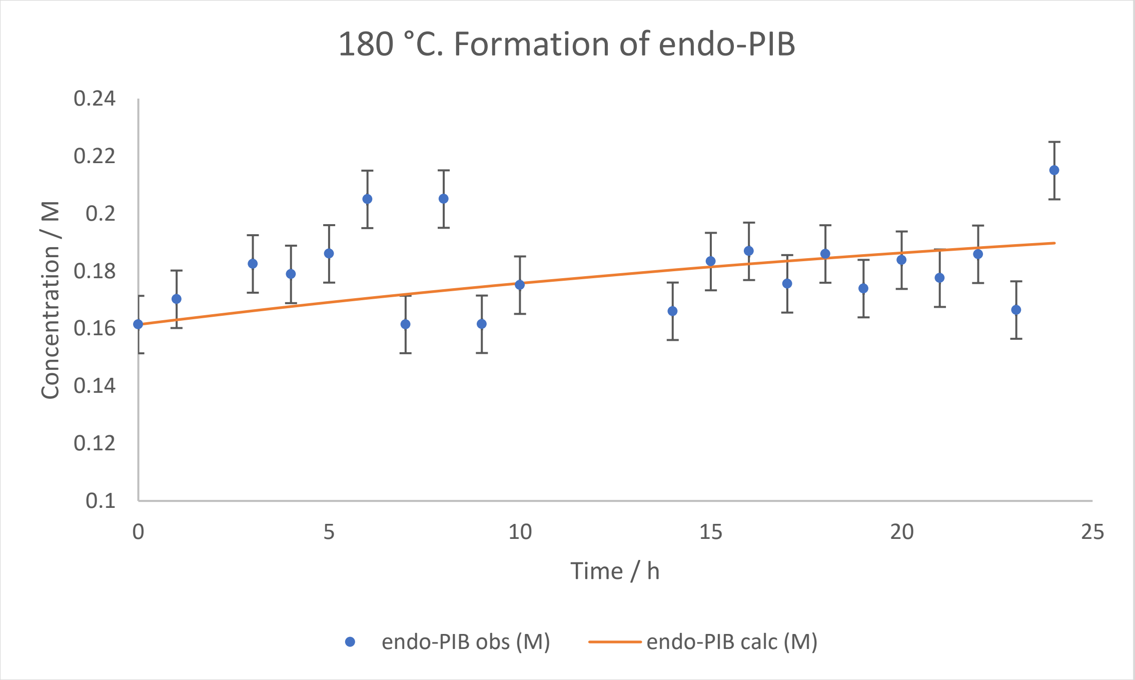

Figure S32. [*endo*-**PIB**] versus time fitted to a 1st order regime at 180 °C, error bars represent 0.01 M.

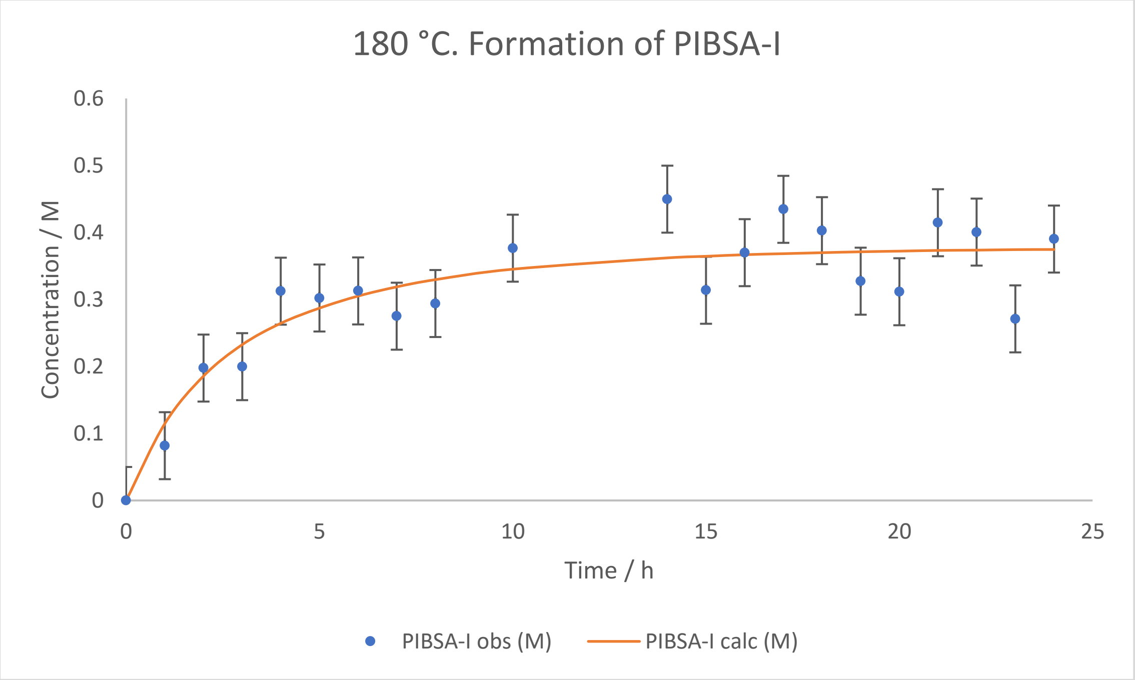

Figure S33. [**PIBSA-I**] versus time fitted to a 2nd order near equal concentrations regime at 180 °C, error bars represent 0.05 M.

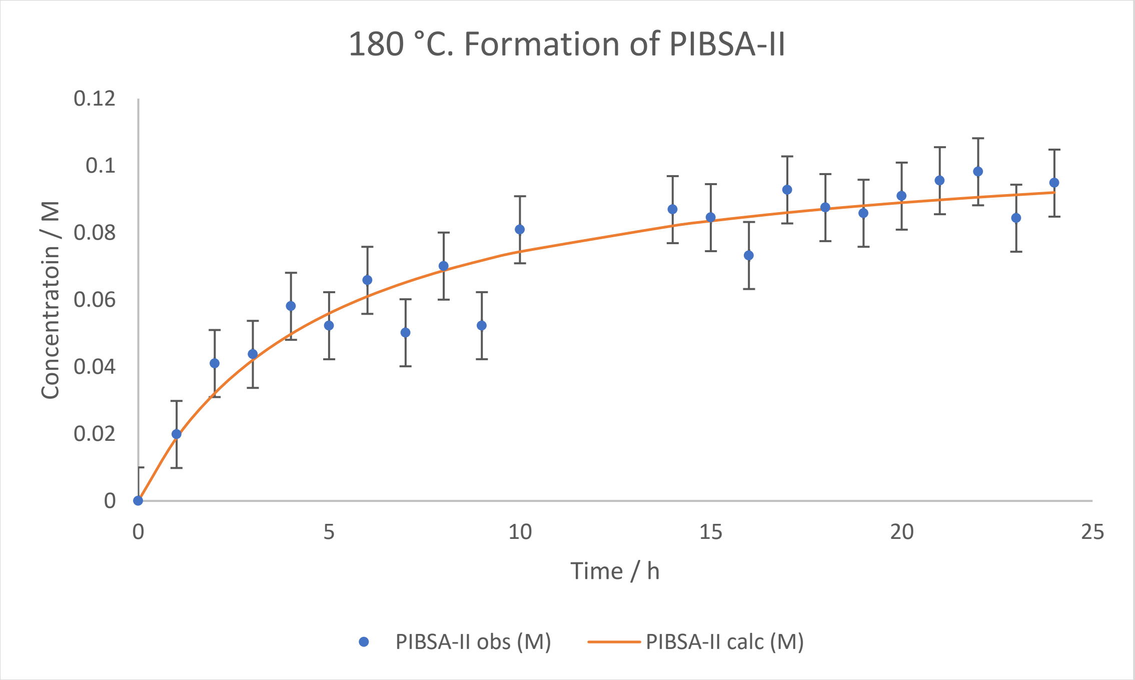

Figure S34. [**PIBSA-II**] versus time fitted to a 2nd order near equal concentrations regime at 180 °C, error bars represent 0.01 M.

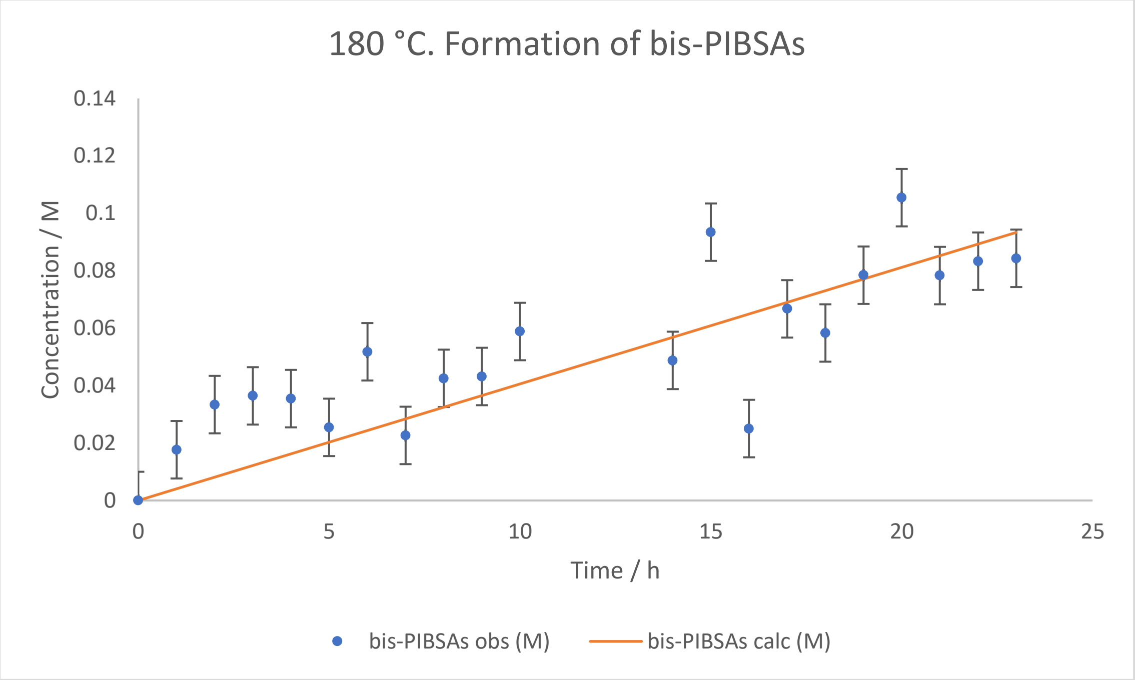

Figure S35. [*bis*-**PIBSAs**] versus time fitted to a 0th order regime at 180 °C, error bars represent 0.01 M.

Table S11. Rate constant data at 180 °C.[a]

Species	k_{obs}[b]	k_1 (s^{-1})	R^2
Consumption of **MAA**	5.0(1) x 10^{-5}	6(1) x 10^{-5}	0.96
Consumption of *exo*-**PIB**	4.1(6) x 10^{-5}	7(1) x 10^{-5}	0.89
Formation of *endo*-**PIB**	1(3) x 10^{-5}	1(3) x 10^{-5}	0.27
Formation of **PIBSA-I**	6(1) x 10^{-5}	1.1(3) x 10^{-4}	0.82
Formation of **PIBSA-II**	6(1) x 10^{-5}	1.0(2) x 10^{-4}	0.92
Formation of *bis*-**PIBSAs**[c]	~4 x 10^{-3}	~2 x 10^{-3}	0.76

[a] Figures in parentheses indicate the estimated standard deviation in the last significant figure.

[b] Units of k_{obs} for **MAA**, *exo*-**PIB**, **PIBSA-I**, and **PIBSA-II** = M^{-1} s^{-1}. Units of k_{obs} for *endo*-**PIB** = s^{-1}. Units of k_{obs} for *bis*-**PIBSAs** = M s^{-1}.

[c] Only obtained in poor fit with large error due to low concentration.

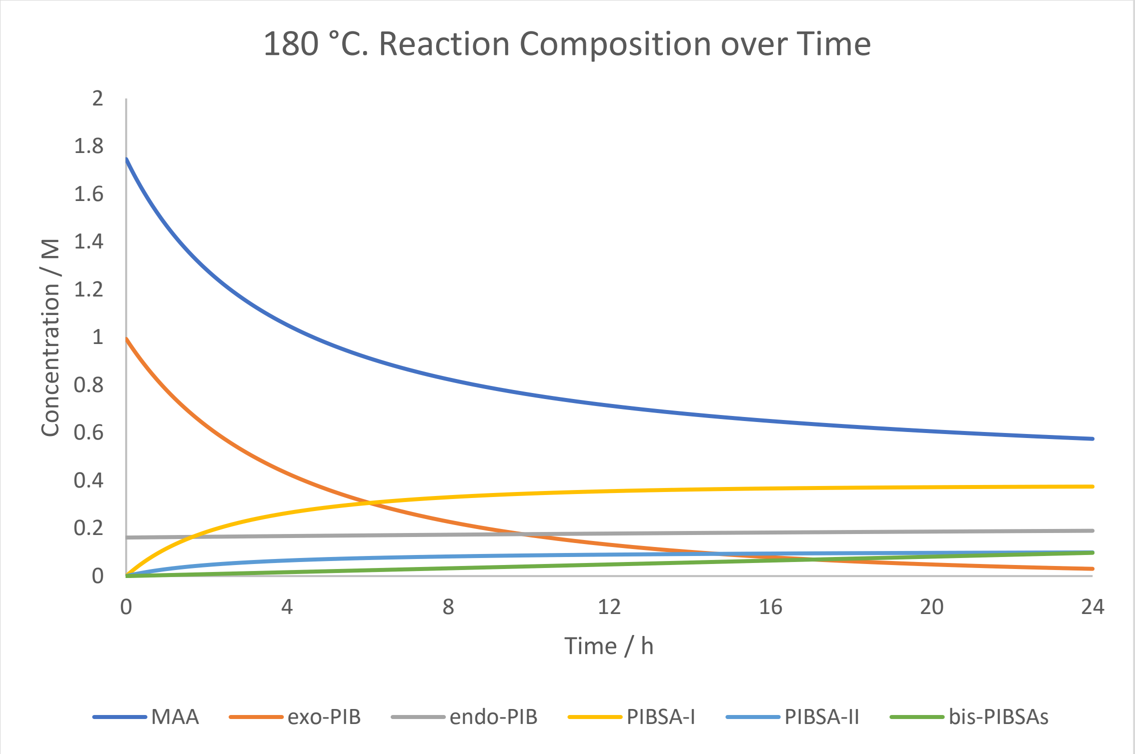

Figure S36. Simulated molarity of species over time at 180 °C using the data of Table S11.

5. Eyring-Polanyi Plots and Derived Reaction Parameters

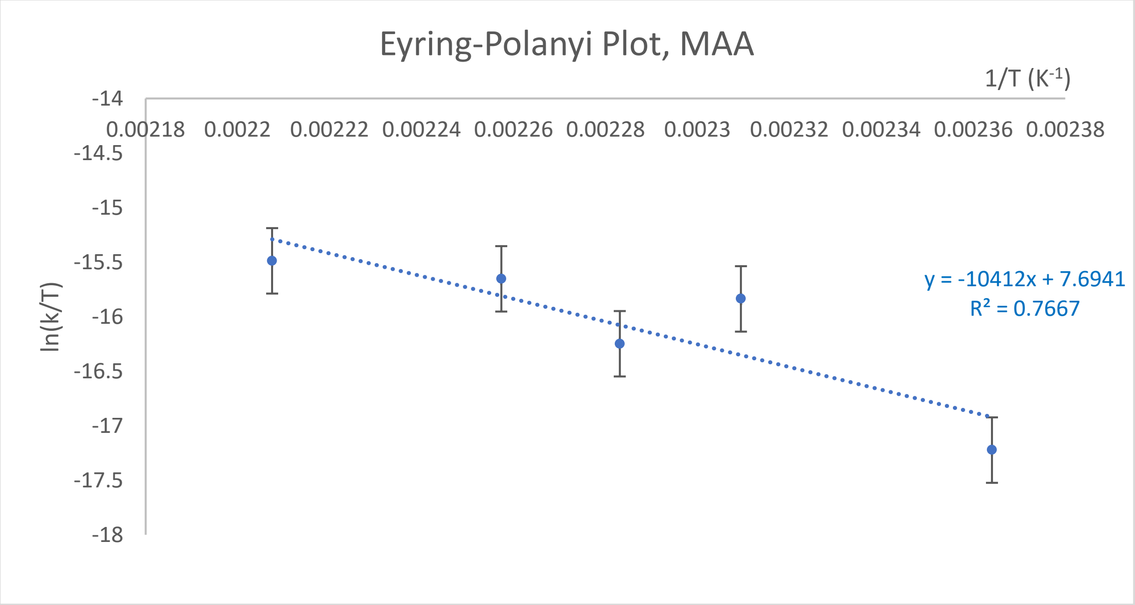

Figure S37. Eyring-Polanyi plot derived from **MAA** dataset. Error bars represent ±0.3 ln(k/T), calculated by converting standard deviation from SolverStat into variance.[7]

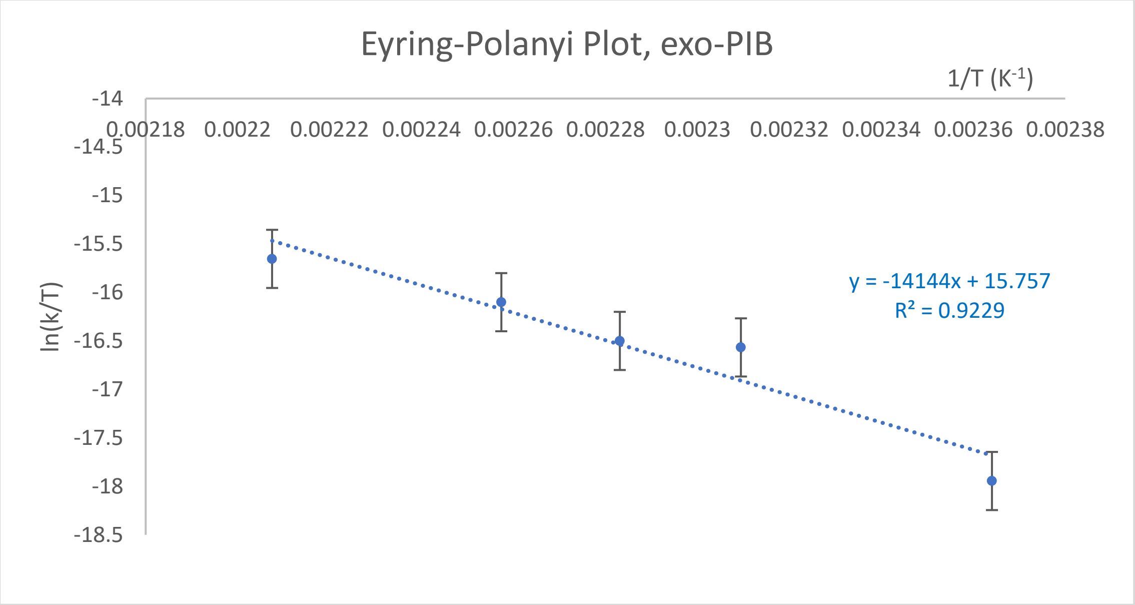

Figure S38. Eyring-Polanyi plot derived from *exo*-**PIB** dataset. Error bars represent ±0.3 ln(k/T), calculated by converting standard deviation from SolverStat into variance.[7]

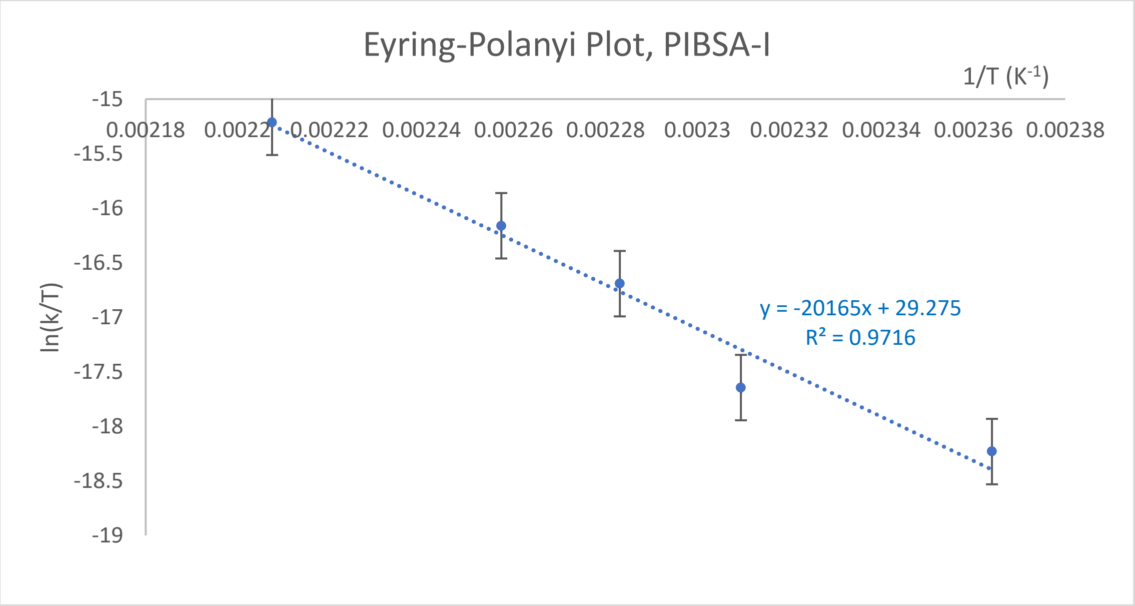

Figure S39. Eyring-Polanyi plot derived from **PIBSA-I** dataset. Error bars represent ±0.3 ln(k/T), calculated by converting standard deviation from SolverStat into variance.[7]

Table S12. Reaction parameters derived from Eyring-Polanyi plots.[a]

			$\Delta G^‡$ (kcal mol^{-1})				
	$\Delta H^‡$ (kcal)	$\Delta S^‡$ (eu)	150 °C	160 °C	165 °C	170 °C	180 °C
From **MAA**	21(7)	-32(15)	34.2 (1.5)	34.5 (1.5)	34.7 (1.6)	34.8 (1.6)	35.2 (1.6)
From *exo*-**PIB**	28(5)	-16(11)	34.8 (2.2)	35.0 (2.2)	35.1 (2.2)	35.2 (2.3)	35.3 (2.3)
From **PIBSA-I**	40(4)	11(9)	35.4 (2.6)	35.3 (2.7)	35.3 (2.7)	35.2 (2.8)	35.1 (2.8)

[a] Figures in parentheses indicate the estimated standard deviation in the last significant figure.

6. Arrhenius Plots and Calculating Reaction Parameters

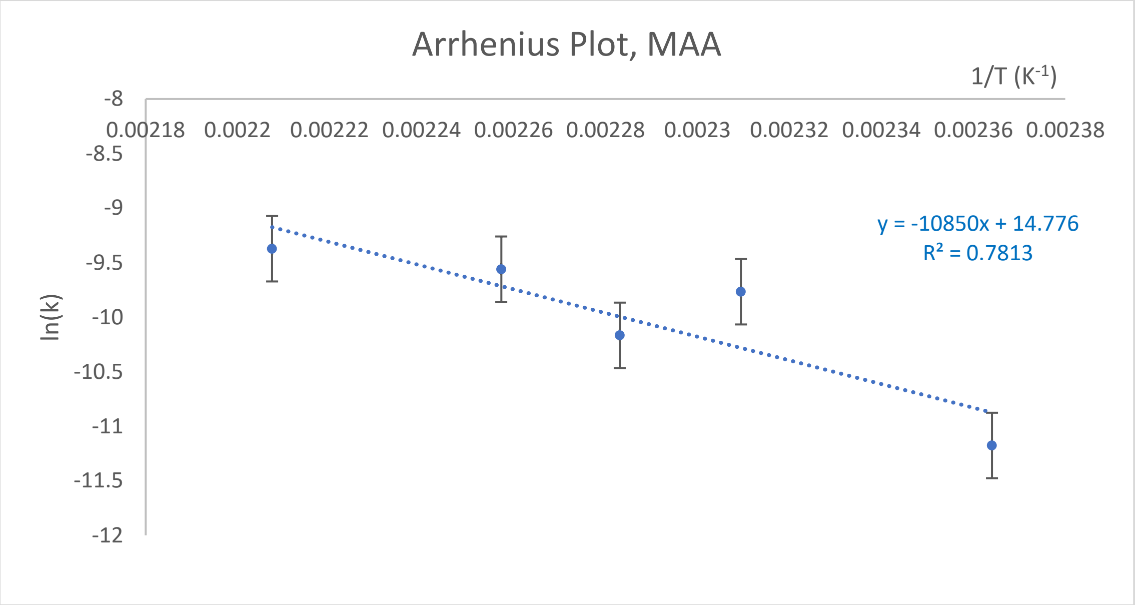

Figure S40. Arrhenius plot derived from **MAA** dataset. Error bars represent ±0.3 ln(k), calculated by converting standard deviation from SolverStat into variance.[7]

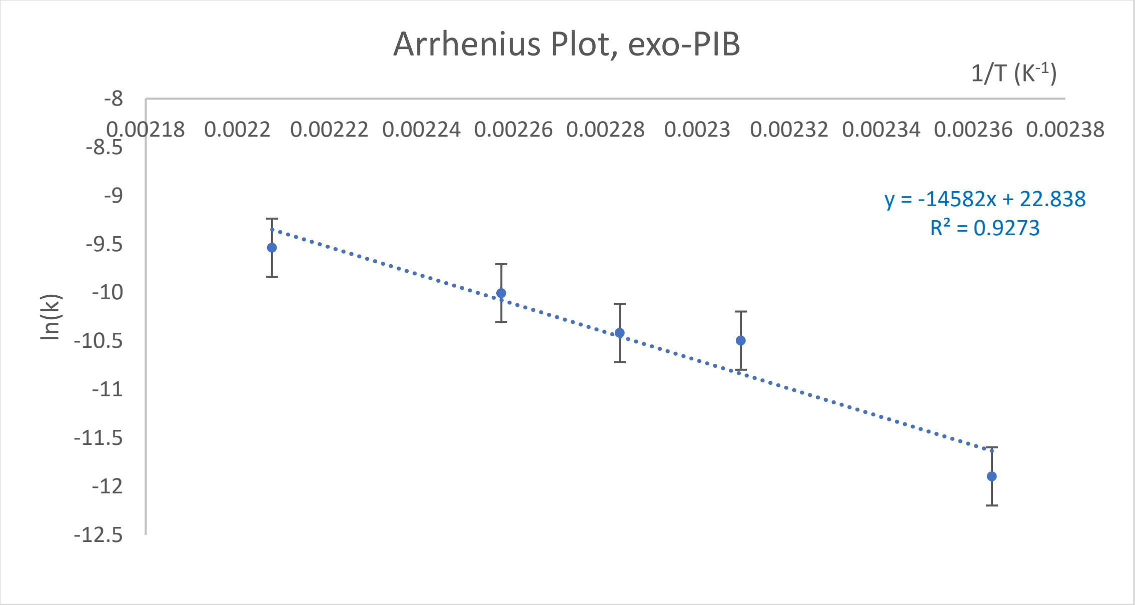

Figure S41. Arrhenius plot derived from *exo*-**PIB** dataset. Error bars represent ±0.3 ln(k), calculated by converting standard deviation from SolverStat into variance.[7]

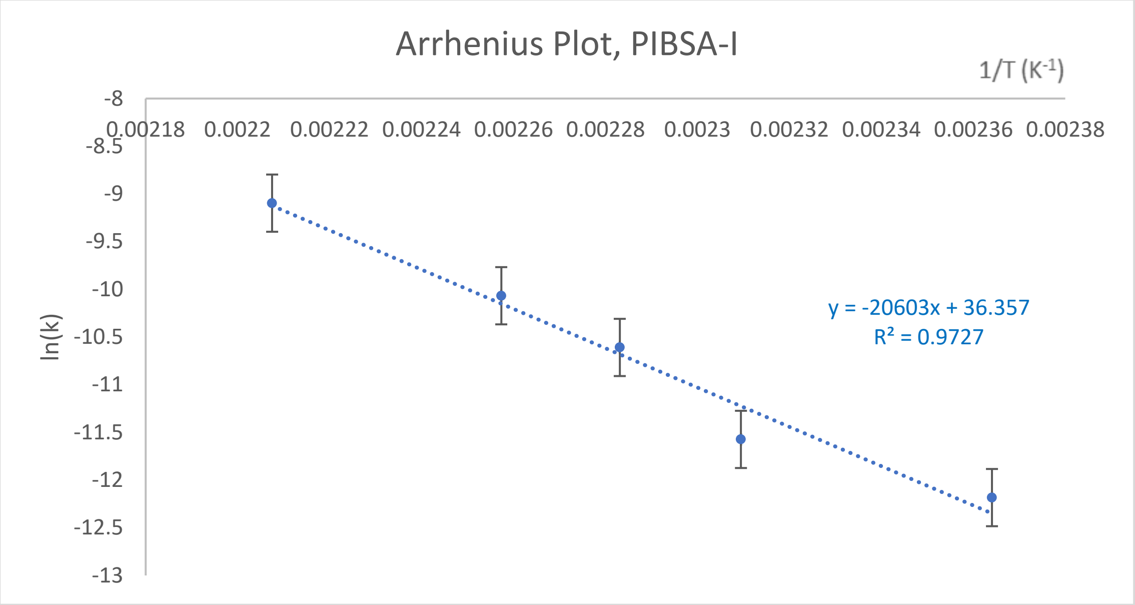

Figure S42. Arrhenius plot derived from **PIBSA-I** dataset. Error bars represent ±0.3 ln(k), calculated by converting standard deviation from SolverStat into variance.[7]

Table S13. Activation energies calculated from Arrhenius plots.

	E_a (kcal mol^{-1})
From **MAA**	21.6(6.6)
From *exo*-**PIB**	29.0(4.7)
From **PIBSA-I**	40.9(4.0)

7. $AlCl_3$ and Quinol Results

Table S14. Reaction after 4 h at 150 °C with $AlCl_3$[a] or $AlCl_3$ and 2mol% quinol

$AlCl_3$ loading (mol%)	*exo*-**PIB** conc. (M)	*endo*-**PIB** conc. (M)	**PIBSA-I** conc. (M)	**PIBSA-II** conc. (M)
3	3.895	1.354	0.188	0.075
3	3.799	1.322	0.191	0.071
3	3.746	1.097	0.161	0.067
5	3.220	1.668	0.184	0.097
5	3.421	1.394	0.190	0.076
5	2.944	1.868	0.158	0.079
8	4.078	2.247	0.250	0.104
8	3.900	1.093	0.101	0.054
8	3.266	1.685	0.135	0.075
5 + 2mol% quinol	3.739	1.391	0.165	0.070
5 + 2mol% quinol	3.306	1.710	0.164	0.071
5 + 2mol% quinol	3.593	1.303	0.1461	0.0974

[a] Screening of >10 alternative Lewis acids species gave no improvement over $AlCl_3$.

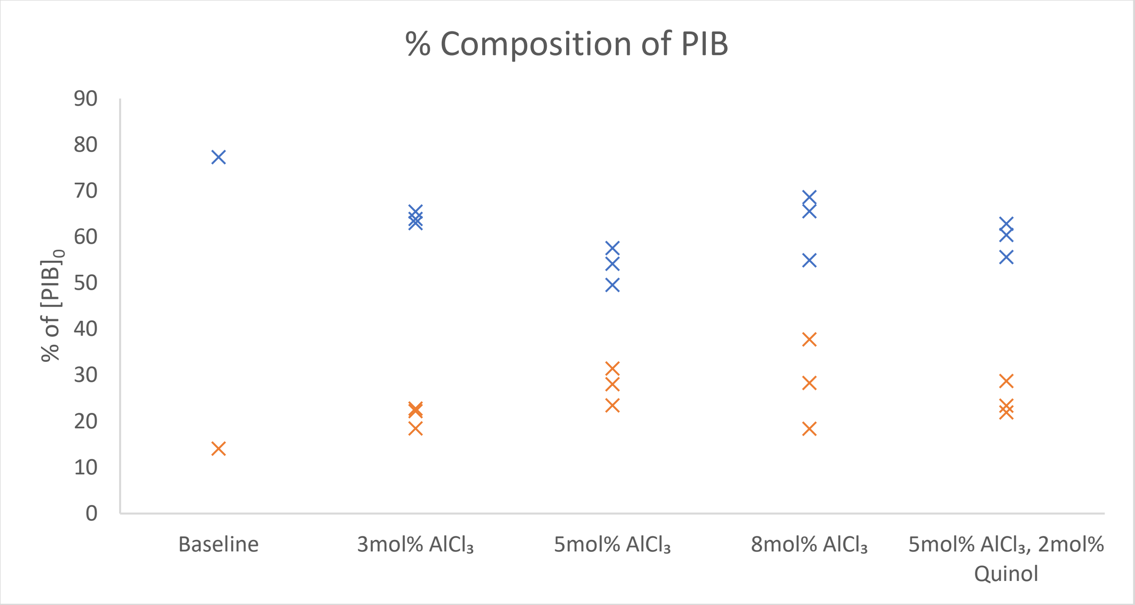

Figure S43. Proportion of *exo*-**PIB** (blue) and *endo*-**PIB** (orange) after 4 h reaction at 150 °C with 3-8mol% $AlCl_3$ and 5mol% $AlCl_3$ and 2mol% quinol.

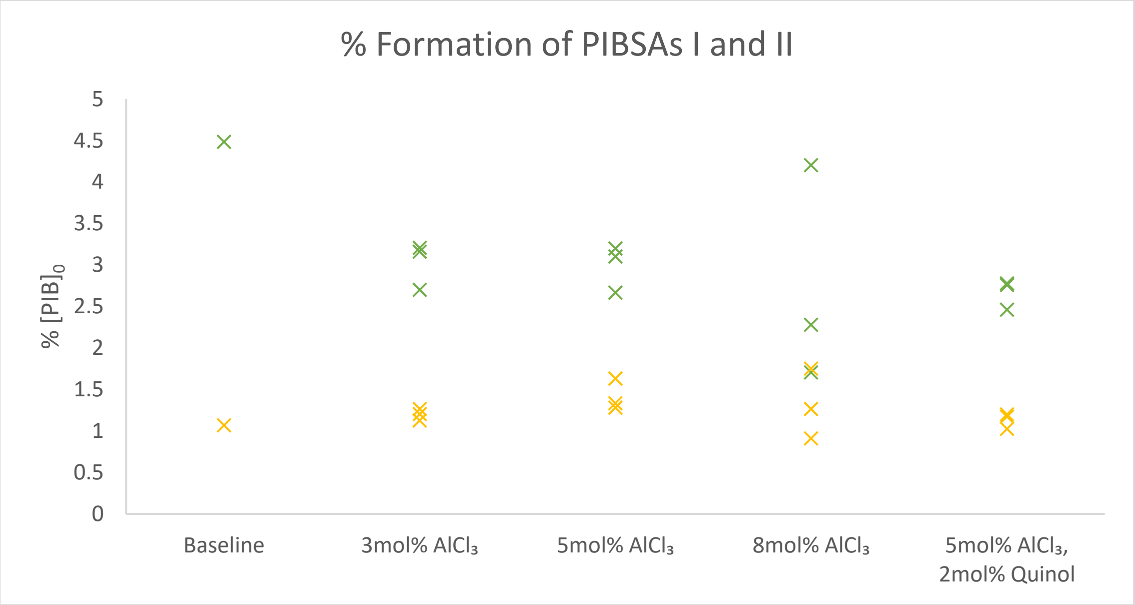

Figure S44. Proportion of **PIBSA-I** (green) and **PIBSA-II** (yellow) after 4 h reaction at 150 °C with 3-8mol% $AlCl_3$ and 5mol% $AlCl_3$ and 2mol% quinol.

Table S15. Reaction at 165 °C with 2% quinol.

Time / h	*exo*-**PIB** conc. (M)	**MAA** conc. (M)
0	1.241	1.746
3	0.810	1.253
6	0.579	0.987
9	0.644	0.606
15	0.320	0.615
18	0.428	0.680
21	0.110	0.535

24	0.157	0.316

Table S16. Rate constant data at 165 °C with 2% quinol.[a]

Species	k_{obs}[b]	k_1 (s^{-1})	R^2
Consumption of *exo*-**PIB**, baseline	1.7(2) x 10^{-5}	3.0(4) x 10^{-5}	0.87
Consumption of *exo*-**PIB**, 2% quinol	1.4(4) x 10^{-5}	2.5(7) x 10^{-5}	0.81

[a] Figures in parentheses indicate the estimated standard deviation in the last significant figure.

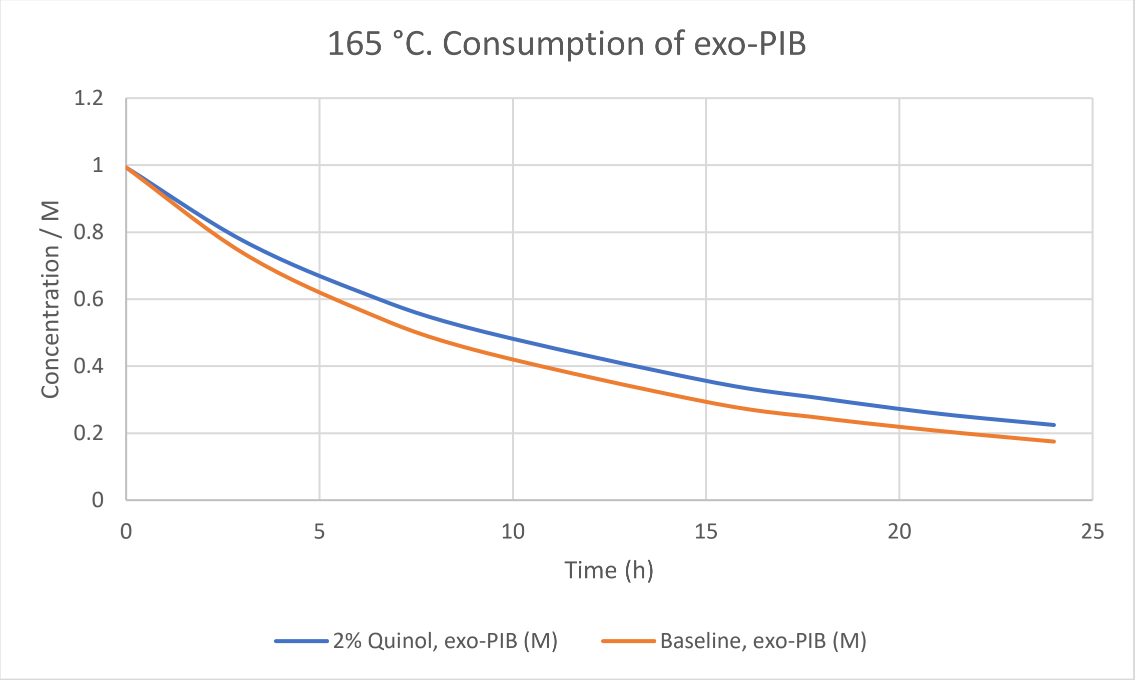

Figure S45. [*exo*-**PIB**] versus time fitted to a 2nd order near equal concentrations regime at 165 °C, standard reaction conditions (orange) and containing 2% quinol (blue).

8. References

(1) Balzano, F.; Pucci, A.; Rausa, R.; Uccello-Barretta, G. Alder-Ene Addition of Maleic Anhydride to Polyisobutene: Nuclear Magnetic Resonance Evidence for an Unconventional Mechanism. *Polym. Int.* **2012**, *61* (8), 1256–1262. https://doi.org/10.1002/pi.4228.

(2) Martuano, R. The Kinetics and Mechanism of the Ene Reaction of Vinylidene Alkenes with Maleic Anhydride, University of Sussex, UK, 2001.

(3) Hossain, M. D.; Ngo, H.; Guo, W. Introductory of Microsoft Excel SOLVER Function-Spreadsheet Method for Isotherm and Kinetics Modelling of Metals Biosorption in Water and Wastewater. *J. Water Sustain.* **2013**, *3* (4), 223–237.

(4) Adekunbi, E. A.; Babajide, J. O.; Oloyede, H. O.; Amoko, J. S.; Obijole, O. A.; Oke, I. A. Evaluation of Microsoft Excel Solver as a Tool for Adsorption Kinetics Determination. *Ife J. Sci.* **2020**, *21* (3), 169–183. https://doi.org/10.4314/ijs.v21i3.14.

(5) Harris, D. C. Nonlinear Least-Squares Curve Fitting with Microsoft Excel Solver. *J. Chem. Educ.* **1998**, *75* (1), 119–121. https://doi.org/10.1021/ed075p119.

(6) Espenson, J. H. *Chemical Kinetics and Reaction Mechanisms*; McGraw-Hill, 1981.

(7) Comuzzi, C.; Polese, P.; Melchior, A.; Portanova, R.; Tolazzi, M. SOLVERSTAT: A New Utility for Multipurpose Analysis. An Application to the Investigation of Dioxygenated Co(II) Complex Formation in Dimethylsulfoxide Solution. *Talanta* **2003**, *59* (1), 67–80. https://doi.org/10.1016/S0039-9140(02)00457-5.
